# Spectroscopic and biochemical insight into an electron-bifurcating [FeFe] hydrogenase

**DOI:** 10.1007/s00775-019-01747-1

**Published:** 2019-12-10

**Authors:** Nipa Chongdar, Krzysztof Pawlak, Olaf Rüdiger, Edward J. Reijerse, Patricia Rodríguez-Maciá, Wolfgang Lubitz, James A. Birrell, Hideaki Ogata

**Affiliations:** 1 Max Planck Institute for Chemical Energy Conversion, Stiftstrasse 34-36, 45470 Mülheim an der Ruhr, Germany; 2 Institute of Low Temperature Science, Hokkaido University, Kita-19, Nishi-8, Kita-ku, Sapporo, 060-0819 Japan

**Keywords:** [FeFe] hydrogenase, Electron bifurcation, Spectroscopy, Electrochemistry, Ferredoxin

## Abstract

**Abstract:**

The heterotrimeric electron-bifurcating [FeFe] hydrogenase (HydABC) from *Thermotoga maritima* (*Tm*) couples the endergonic reduction of protons (H^+^) by dihydronicotinamide adenine dinucleotide (NADH) (∆*G*^0^ ≈ 18 kJ mol^−1^) to the exergonic reduction of H^+^ by reduced ferredoxin (Fd_red_) (∆*G*^0^ ≈ − 16 kJ mol^−1^). The specific mechanism by which HydABC functions is not understood. In the current study, we describe the biochemical and spectroscopic characterization of *Tm*HydABC recombinantly produced in *Escherichia coli* and artificially maturated with a synthetic diiron cofactor. We found that *Tm*HydABC catalyzed the hydrogen (H_2_)-dependent reduction of nicotinamide adenine dinucleotide (NAD^+^) in the presence of oxidized ferredoxin (Fd_ox_) at a rate of  ≈17 μmol NADH min^−1^ mg^−1^. Our data suggest that only one flavin is present in the enzyme and is not likely to be the site of electron bifurcation. FTIR and EPR spectroscopy, as well as FTIR spectroelectrochemistry, demonstrated that the active site for H_2_ conversion, the H-cluster, in *Tm*HydABC behaves essentially the same as in prototypical [FeFe] hydrogenases, and is most likely also not the site of electron bifurcation. The implications of these results are discussed with respect to the current hypotheses on the electron bifurcation mechanism of [FeFe] hydrogenases. Overall, the results provide insight into the electron-bifurcating mechanism and present a well-defined system for further investigations of this fascinating class of [FeFe] hydrogenases.

**Graphic abstract:**

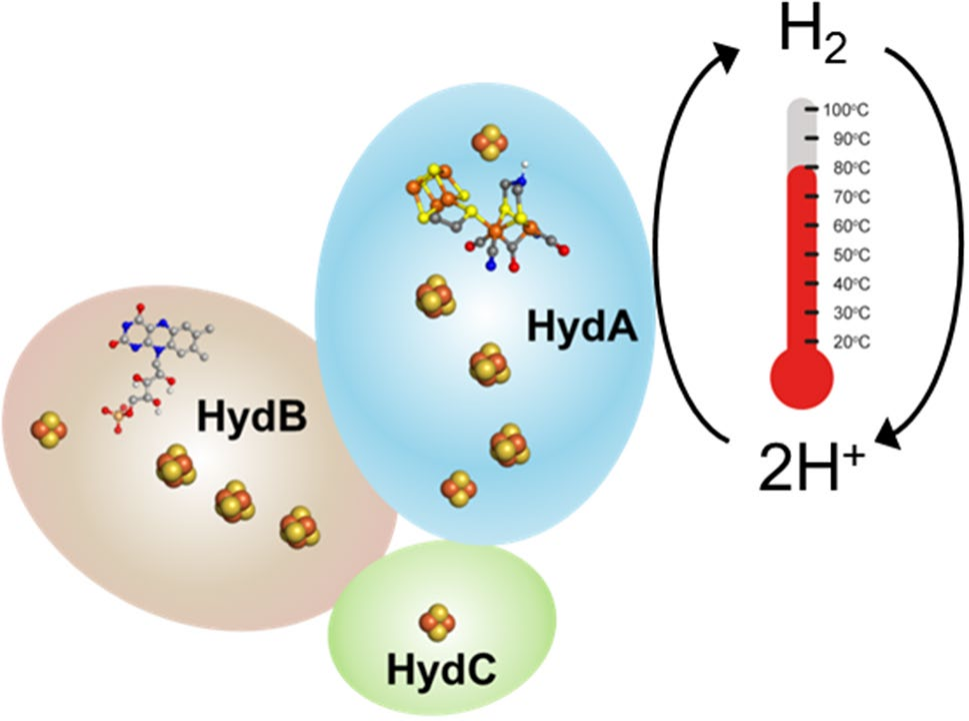

**Electronic supplementary material:**

The online version of this article (10.1007/s00775-019-01747-1) contains supplementary material, which is available to authorized users.

## Introduction

**Fig. 1:**
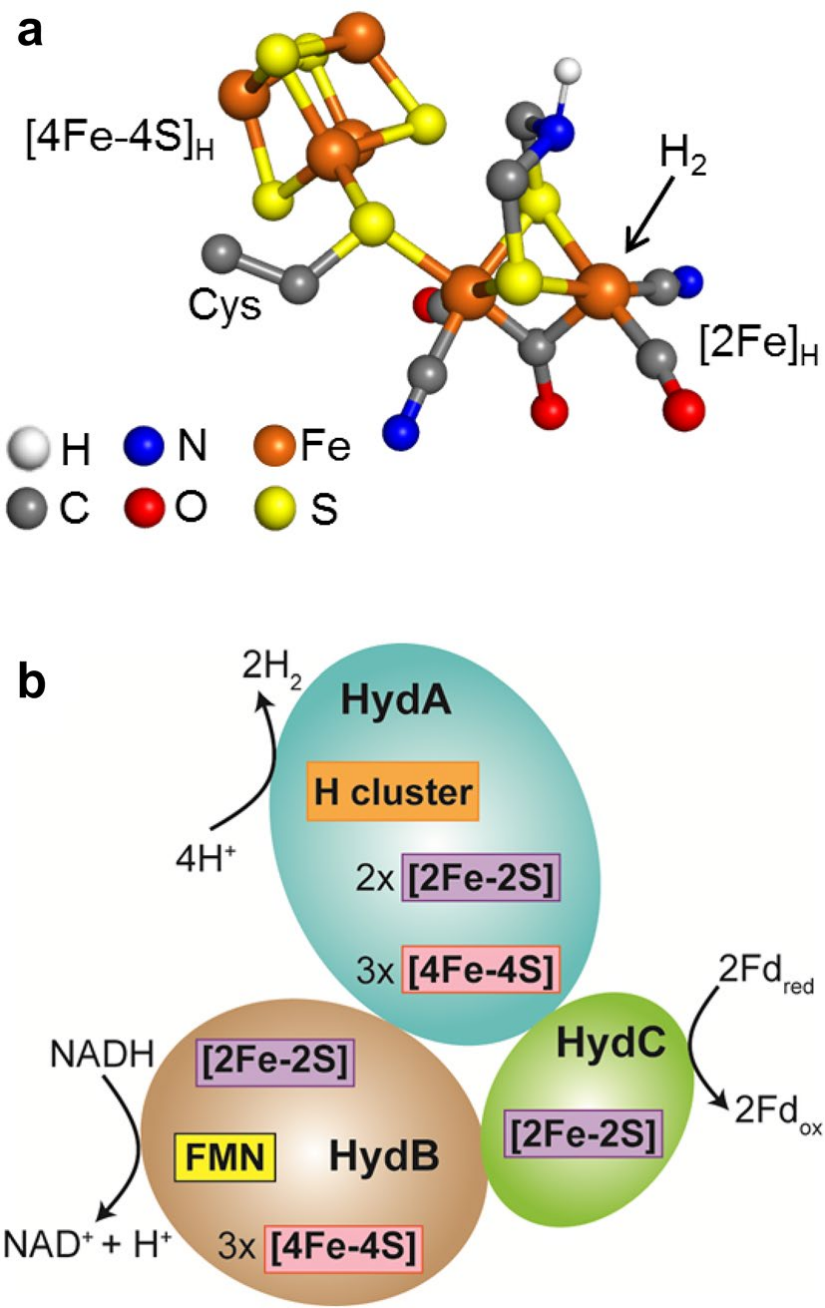
**a** Ball and stick representation of the structure of the H-cluster. The figure was created in Pymol using PDB file 4XDC. **b** Schematic diagram showing the three subunits of *Tm*HydABC. The cofactors bound to the protein were predicted from amino acid sequence analysis [26]. The arrangement of subunits and the designated reactions occurring at each subunit are based on the proposition by Buckel and Thauer [23]

[FeFe] hydrogenases catalyze the reversible interconversion of protons (H^+^) and electrons to hydrogen (H_2_) at very high rates, with negligible energy waste [1–3]. Their active center, the H-cluster, consists of a unique [2Fe] cluster ([2Fe]_H_) tethered covalently by a cysteine thiolate to a standard [4Fe–4S] cluster ([4Fe–4S]_H_) (Fig. 1a) [4, 5]. The Fe ions in the [2Fe]_H_ subsite are coordinated by CO and CN^−^ ligands and also by a bidentate 2-azapropane 1,3-dithiolate (ADT) ligand that bridges the two irons of [2Fe]_H_ (Fig. 1a). The H-cluster is buried inside a highly optimized protein scaffold, which tunes its catalytic efficiency and provides pathways for the transport of protons, electrons, and gases (the substrate H_2_ as well as inhibitors such as CO and O_2_) [6].

During H_2_ conversion, the H-cluster passes through several redox states [1]. In the active oxidized state (H_ox_), the [2Fe]_H_ subcluster is in a mixed valent [Fe(I)Fe(II)] state, and the [4Fe–4S]_H_ subsite is oxidized (2 +). One-electron reduction of the [4Fe–4S]_H_ subsite forms the H_red_ state [7]. Protonation of the ADT in the H_red_ state is coupled to electron transfer from the reduced [4Fe–4S]_H_ to the [2Fe] cluster, yielding the H_red_H^+^ state [8]. A second electron reduction forms the most reduced state, H_sred_H^+^ ([4Fe–4S]^1+^–[Fe(I)Fe(I)]) [9]. Rearrangement of the H_sred_H^+^ state gives the H_hyd_ state, where a terminal hydride is bound to the [2Fe] subsite with an Fe(II)Fe(II) configuration and [4Fe–4S]_H_ remains reduced [10, 11]. Protonation of H_hyd_ leads to H_2_ production and regeneration of the H_ox_ state.

Based on amino acid sequence phylogeny, the [FeFe] hydrogenases have been classified into three major groups: (I) prototypical and electron-bifurcating; (II) ancestral; and (III) sensory [12]. The physiological roles of the prototypical [FeFe] hydrogenases are far better understood than the other classes. While the prototypical [FeFe] hydrogenases use ferredoxin or cytochrome as their sole redox partner, the electron-bifurcating [FeFe] hydrogenases use both ferredoxin (Fd) and dihydronicotinamide adenine dinucleotide (NADH) simultaneously during H_2_ evolution [13–16].

Electron bifurcation is a process, whereby an endergonic redox reaction and an exergonic redox reaction are directly coupled, and is an alternative energy conservation mechanism to the well-known chemiosmotic coupling mechanism [16, 17]. First described by Peter Mitchell in the Q-cycle of mitochondrial complex III [18], it was only about a decade ago that the involvement of electron bifurcation in the metabolism of anaerobic microorganisms was discovered [19]. Subsequent investigations showed that in a variety of reactions in anaerobic metabolism, electron bifurcation is involved [20], among them: coupling of the endergonic oxidation of NADH (*E*_0_′ = − 320 mV) to the exergonic oxidation of reduced ferredoxin (Fd_red_) (*E*_0_′ ≈ − 450 mV) to reduce H^+^ to H_2_ (*E*_0_′ = − 420 mV). The overall reaction is the recycling of NADH, generated by the oxidation of sugars, driven by Fd_red_ oxidation and can be described in full as [14, 16, 21–23]:

1 $${\text{NADH}} + 2{\text{Fd}}_{{{\text{red}}}} + 3{\text{H}}^{ + } \rightleftharpoons {\text{NAD}}^{ + } + 2{\text{Fd}}_{{{\text{ox}}}} + 2{\text{H}}_{2}$$

This reaction is essential in many organisms as various metabolic steps only yield enough energy to reduce nicotinamide adenine dinucleotide (NAD^+^), but NADH is not capable of reducing protons. Thus, by coupling NADH oxidation to ferredoxin oxidation, protons can be used as a terminal electron acceptor [20, 21].

Electron-bifurcating [FeFe] hydrogenases are widely found in the genomes of anaerobic bacteria belonging to the phyla of *Bacteroidetes*, *Firmicutes*, *Spirochaetes*, *Thermotogae,* and *Fusobacteria* [12]. Bacteria belonging to *Bacteroidetes* and *Firmicutes* phyla are commonly found in mammalian guts, and as H_2_ is an abundant metabolite in the gastrointestinal tract, hydrogenases found in the above-mentioned organisms play an important role in colonic H_2_ metabolism [24]. Interestingly, in a recent bioinformatics study, it has been shown that a wide variety of hydrogenases found in the bacteria of the human gut belong to the electron-bifurcating class [25]. Therefore, understanding the metabolism of these organisms may also be important for medical applications.

Electron-bifurcating [FeFe] hydrogenases have been characterized from a number of organisms; however, the main focus of all these studies were their biochemical properties [14, 21, 27–29]. Even though the electron-bifurcating [FeFe] hydrogenase from *Thermotoga maritima* (*Tm*) has been spectroscopically characterized [26, 30], ambiguities in the understanding of its biophysical properties still persist. In this work, based on the previous efforts of recombinant expression and artificial maturation of [FeFe] hydrogenases, we have developed a recombinant system to overexpress *Tm*HydABC in *E. coli* followed by its artificial maturation in vitro. This system allows the generation of high yields of pure enzyme, making it possible to perform a complete spectroscopic analysis to study in detail the electron-bifurcating mechanism of *Tm*HydABC [FeFe] hydrogenase.

The genome of the hyperthermophilic bacterium, *T. maritima*, contains genes encoding a prototypical, a sensory, and an electron-bifurcating type [FeFe] hydrogenase [14, 21, 31]. Previous biochemical studies showed that the electron-bifurcating [FeFe] hydrogenase, *Tm*HydABC, is the key enzyme responsible for H_2_ production in *T. maritima* [14]. The electron-bifurcating [FeFe] hydrogenase from *T. maritima* is a trimeric protein composed of *Tm*HydA, *Tm*HydB, and *Tm*HydC (Fig. 1b) [26]. The largest subunit, *Tm*HydA, harbors the H-cluster along with three additional [4Fe–4S] and two [2Fe–2S] clusters. *Tm*HydB is the second largest subunit and consists of three [4Fe–4S] clusters, one [2Fe–2S] cluster, a flavin mononucleotide (FMN) binding domain, and an NADH-binding motif. *Tm*HydC is the smallest of the three subunits and contains only one [2Fe–2S] cluster [32, 33].

The mechanistic study of prototypical [FeFe] hydrogenases has greatly benefited from the discovery of ‘artificial maturation’, whereby heterologously produced apo-proteins, that is the protein containing the [4Fe–4S]_H_ sub cluster but lacking [2Fe]_H_, have been reconstituted using chemically synthesized precursor complexes [34, 35]. Here, we have expanded the repertoire of hydrogenases to which artificial maturation can be applied by employing it to produce the trimeric *Tm*HydABC and also the catalytic subunit *Tm*HydA. Subsequently, we have performed biochemical assays with the resulting hydrogenase as well as a full spectroscopic characterization of the H-cluster. The results reveal insight into the bifurcation mechanism and even more importantly provide the basis for further investigation of this unresolved phenomenon.

## Materials and methods

### Activity assays

Hydrogen oxidation activity of *Tm*HydABC and *Tm*HydA was measured by following the reduction of 1 mM benzyl viologen (at 600 nm, *ε*_600_ = 7.8 mM^−1^ cm^−1^) in 200 mM H_2_-saturated potassium phosphate buffer, pH 8. The reactions were performed at various temperatures (30–80 °C) by the addition of 25–50 ng protein to 1 mL of the above-mentioned reaction buffer in 1.5 mL plastic cuvettes and the change in absorbance was measured using an Ocean Optics DH-mini UV–Vis–NIR light source and a USB2000 + XR1-ES detector, operated by the SpectraSuite^TM^ software. The desired reaction temperatures were achieved using a temperature controlled cuvette holder (CUV-QPOD-2E-ABSKIT, Ocean Optics). All values are the average of three measurements after subtracting the value of the blank measurement. The other details are described in the figure captions.

The NAD^+^ and ferredoxin-dependent H_2_ oxidation assay of *Tm*HydABC was performed at 70 °C by addition of 300–600 ng of the protein to 1 mL of H_2_ saturated 200 mM potassium phosphate buffer, pH 8. The formation of NADH (monitored at 340 nm, *ε* = 6.2 mM^−1^ cm^−1^) and reduction of ferredoxin (monitored at 430 nm, *ε* = 12 mM^−1^ cm^−1^) were determined from the change in absorbance measured using a spectrophotometer.

To determine the methyl viologen dependent hydrogen production, 250–500 ng of protein was added to 10 mM methyl viologen reduced with 100 mM sodium dithionite (NaDT) in 200 mM potassium phosphate buffer pH 8 in 2.5 mL plastic tubes with rubber stoppers. The reaction mixture was purged with argon for 5 min and incubated at the desired reaction temperature for 10 min before 0.5 mL of the headspace gas was extracted for analysis. The head space gas was then analyzed by gas chromatography. Hydrogen content was quantified by comparison with a 100% H_2_ standard. All values are the average of three measurements after subtracting the value of a blank measurement.

### Electrochemistry

Protein film electrochemistry experiments were performed using a gas-tight three electrode setup inside an anaerobic glovebox (MBraun) filled with N_2_. The cell temperature was controlled by a water-jacket system. A pyrolytic graphite edge disk (0.03 cm^2^, Momentive Materials) was used as a working electrode and a Pt wire as a counter electrode. The reference electrode (saturated calomel electrode—SCE) was placed in a side arm (at room temperature), connected to the main cell compartment by a Luggin capillary. Electrochemical experiments were controlled by a VersaStat 4 potentiostat (Princeton Applied Research). The electrochemical cell was filled with a buffer mixture of MES, HEPES, TAPS, CHES, and sodium acetate adjusted to pH 5, 6, 7, 8, 9, and 10. Cyclic voltammetry was performed under 100% H_2_ with a 2000 rpm rotation rate (Princeton Applied Research model 636A). The potentials were converted to the standard hydrogen electrode (SHE) using a conversion of + 241 mV from SCE.

### FTIR spectroscopy

FTIR spectroscopy was performed using home-built water-cooled sample holders accommodated in a Bruker Vertex v80 spectrometer. Samples (10 μL) were placed between two CaF_2_ (Korth Kristalle, Altenholz) windows, separated by a 50 μm Teflon spacer, and sealed with rubber rings. The temperature of the sample holder was maintained using a water circulator system (Huber, Offenburg). The spectrometer was equipped with a liquid nitrogen cooled fast mercury cadmium telluride photovoltaic (D317/B) detector. Spectra were collected at room temperature in double-sided, forward–backward mode with a 2 cm^−1^ resolution and 20 kHz scanner velocity.

### FTIR spectroelectrochemistry

Spectroelectrochemical experiments were performed using a home-built electrochemical cell according to the design described by Moss et al. [1, 36]. Protein samples (1–1.5 mM) containing 0.5 mM of the redox mediators benzyl viologen (*E*_m_ = − 358 mV) and methyl viologen (*E*_m_ = − 449 mV) were loaded between two CaF_2_ (Korth Kristalle, Altenholz) windows on a gold mesh working electrode (approximately 20 μm thick) in electrical contact with a platinum counter electrode. An Ag/AgCl (1 M KCl) electrode was used as a reference and was calibrated before and after the measurement with (hydroxymethyl)ferrocene (*E*_*m*_ = + 436 mV). In the titrations, the potential was controlled by an Autolab PGSTAT101 potentiostat controlled by the Nova software.

The measurements were performed using a Bruker IFS 66v/S FTIR spectrometer equipped with a mercury cadmium telluride photovoltaic detector in double-sided, forward–backward mode with 2 cm^−1^ resolution, and 20 kHz scanner velocity. The temperature (15 °C) was maintained by a water circulator (Huber). Spectra were collected after 40 min incubation time at each applied potential.

### EPR spectroscopy

X-band EPR spectra were recorded on a Bruker ELEXSYS E500 CW X-band EPR spectrometer. The temperature of the samples was controlled using an Oxford Instruments ESR900 helium flow cryostat connected to an ITC503 temperature controller. The measurement parameters were: microwave frequency 9.64 GHz, time constant 81.92 ms, conversion time 81.92 ms, and modulation frequency 100 kHz. The microwave power and temperature were varied between measurements and are indicated in the figure legends.

The EPR samples (200 µL) were transferred anaerobically to 4 mm (o.d.) quartz EPR tubes and frozen in liquid nitrogen. All spectra were analyzed with home-written scripts in MATLAB. Spectral simulations were performed using the EasySpin package [37]. Spin quantification was achieved by comparison with a 1 mM CuSO_4_, 10 mM EDTA standard.

## Results

### Characterization of apo-*Tm*HydABC and apo-*Tm*HydA

The apo-*Tm*HydABC and apo-*Tm*HydA were produced recombinantly in *E. coli* (details of construct preparation, heterologous expression, and purification are provided in the Supplementary Information). Iron quantification of apo-*Tm*HydABC and apo-*Tm*HydA indicated the presence of 35 ± 2 and 19 ± 1 moles of iron per mole of protein, respectively, in good agreement with the expected values (36 for apo-*Tm*HydABC and 20 for apo-*Tm*HydA) based on the number of iron–sulfur (FeS) clusters, and on the previously reported data on *Tm*HydABC and *Tm*HydA isolated from the native organism [26]. Flavin quantification gave 0.2 ± 0.05 moles of FMN per mole of protein. Inclusion of riboflavin during protein overexpression did not enhance the FMN content. Thus, it is likely that the majority of the FMN is lost during purification, as has been demonstrated also for native *Tm*HydABC [26].

Apo-*Tm*HydABC and apo-*Tm*HydA were analyzed using UV–Vis and continuous wave (CW) electron paramagnetic resonance (EPR) spectroscopy (Figure S5). In both cases, the UV–Vis spectrum showed broad bands in the range from 300 to 600 nm (Figure S5A) typical for [2Fe–2S] and [4Fe–4S] clusters. EPR spectra from apo-*Tm*HydABC and apo-*Tm*HydA reduced with sodium dithionite are shown in Figure S5B. The EPR spectrum of apo-*Tm*HydABC at 40 K is similar to that observed with the reduced native protein and has *g* values and relaxation properties consistent with [2Fe–2S] clusters (Figure S5B, upper panel) [26]. The EPR spectrum of apo-*Tm*HydABC at 10 K appears to be more complex than that at 40 K, with the broadening of the feature at *g* ≈ 2.02 and appearance of a new feature at *g* ≈ 1.88 (Figure S5B, upper panel). This phenomenon was also observed in the native protein and was attributed to the weak dipolar interactions between the clusters [26]. The EPR spectrum of apo-*Tm*HydA is very similar to the apo-*Tm*HydABC EPR spectrum at 40 K (Figure S5B, lower panel). Interestingly, at 10 K the EPR spectrum of *Tm*HydA does not show such a large contribution from the broad feature as apo-*Tm*HydABC. Nevertheless, it can be concluded that apo-*Tm*HydABC and apo-*Tm*HydA contain both [2Fe–2S] and [4Fe–4S] clusters essentially identical to that of the proteins purified from the native organism.

### Activity assays with artificial partners

**Fig. 2:**
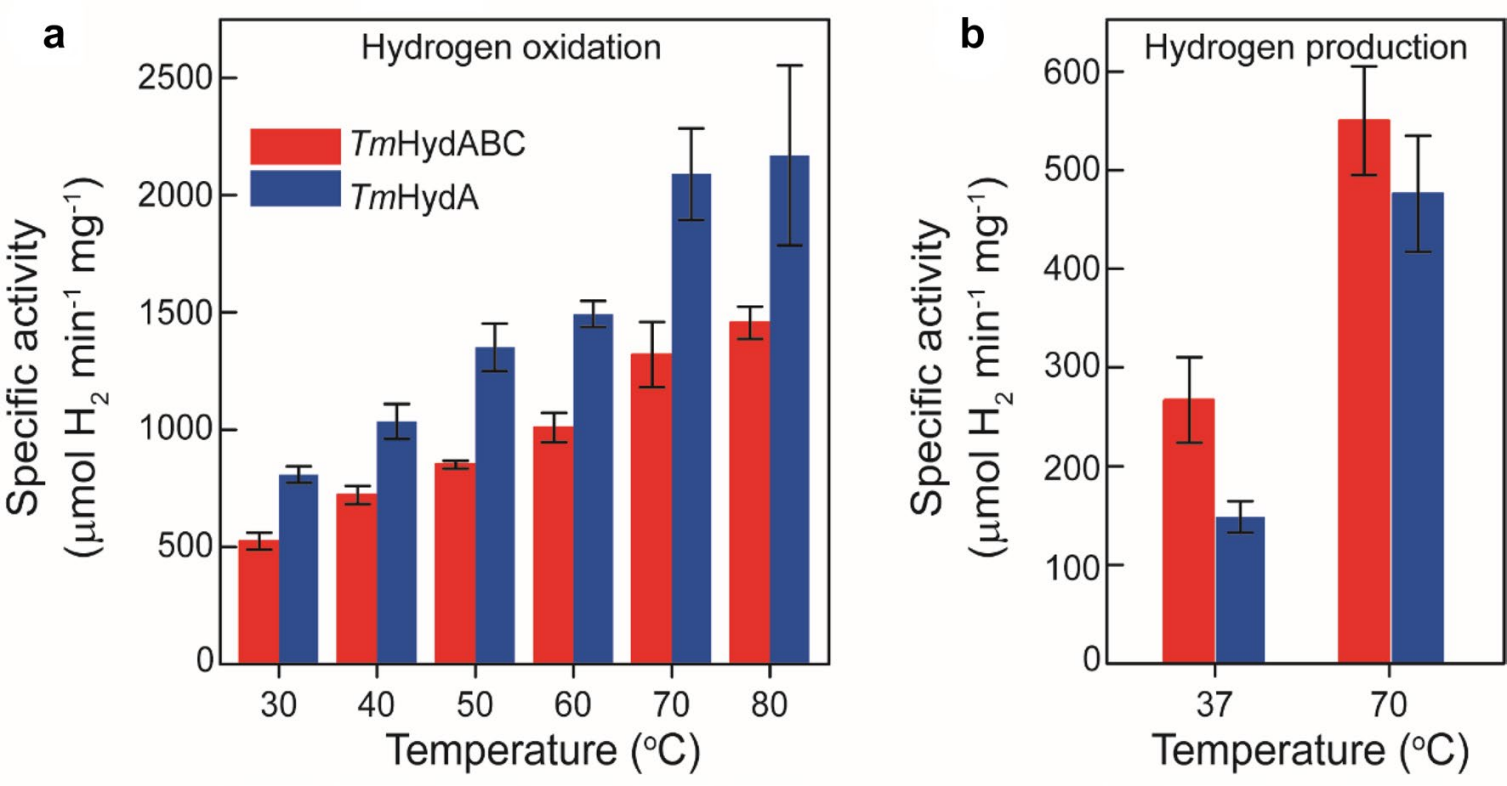
Activity assays of artificially maturated *Tm*HydABC and *Tm*HydA. **a** Temperature dependence of H_2_ oxidation of both proteins was measured in the range from 30 to 80 °C with benzyl viologen as the electron acceptor. **b** H_2_ production activity of *Tm*HydABC and *Tm*HydA was measured at 37 °C and 70 °C with reduced methyl viologen as electron donor

The apo-*Tm*HydABC and apo-*Tm*HydA were artificially maturated using the previously published in vitro method (see Supplementary Text 3 for details) to generate the holo-enzymes [34, 35]. Activity assays with holo-*Tm*HydABC and holo-*Tm*HydA in solution were performed to obtain the rates of H_2_ oxidation coupled to benzyl viologen reduction and H_2_ production coupled to methyl viologen oxidation. At 30 °C, *Tm*HydABC and *Tm*HydA oxidized H_2_ at a rate of ≈500 U mg^−1^ and ≈800 U mg^−1^, respectively (1 U = 1 µmol min^−1^) (Fig. 2a). The H_2_ production activities of *Tm*HydABC and *Tm*HydA at 37 °C were found to be ≈270 U mg^−1^ and ≈150 U mg^−1^, respectively (Fig. 2b). While both activities increased with temperature, a stronger effect was found for H_2_ oxidation (Fig. 2). Compared to *Tm*HydABC isolated from the native organism the artificially maturated protein showed significantly higher activity [38]. This could be related to a higher conversion of the CO-inhibited state H_ox_–CO to the active states in the artificially maturated protein, while in the native organism, a larger fraction of the isolated protein is in the inactive H_ox_–CO state.

### Catalytic activity using protein film electrochemistry

**Fig. 3:**
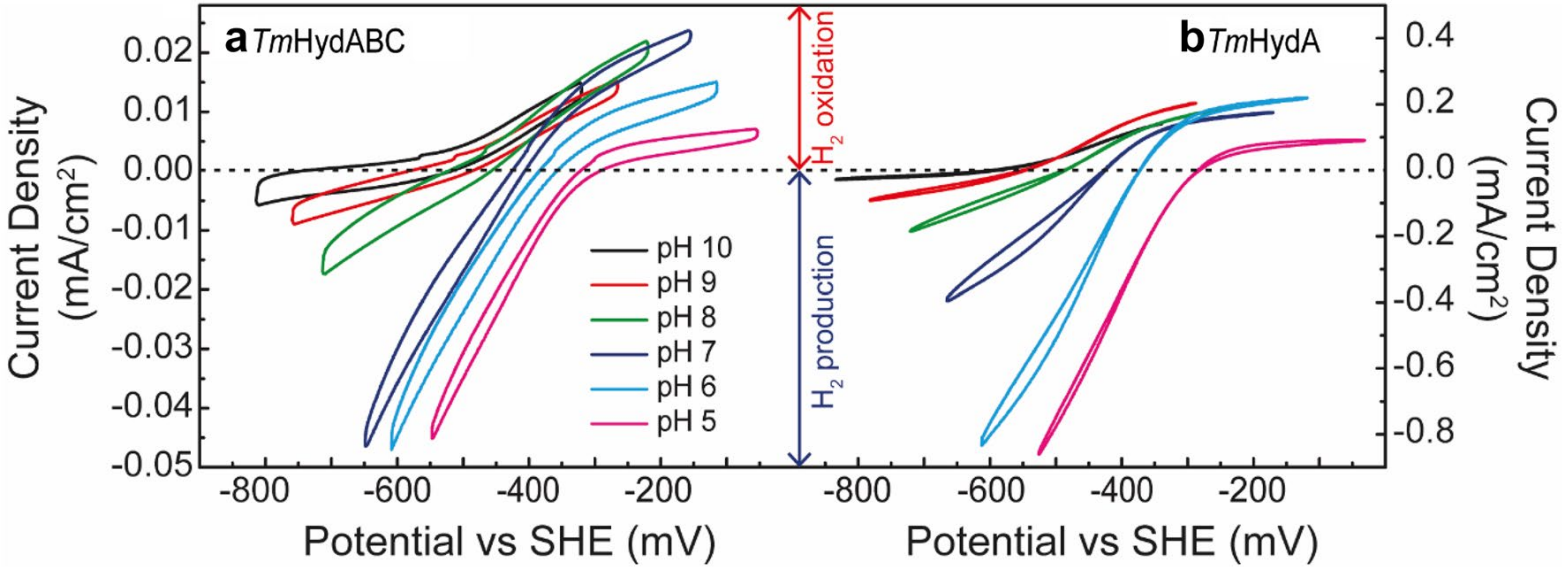
Cyclic voltammetry of *Tm*HydABC (**a**) and *Tm*HydA (**b**) adsorbed onto the surface of a pyrolytic graphite electrode. The applied potential was swept from open-circuit potential to 250 mV more positive and then to 250 mV more negative, to compare the catalytic currents at the same overpotential. The measurements were performed at 35 °C under 100% H_2_

The catalytic activity of *Tm*HydABC and *Tm*HydA was also measured electrochemically by adsorbing the enzymes on pyrolytic graphite electrodes (Fig. 3). Due to protein desorption at increased temperatures, the maximum temperature that could be used was 35 °C. Under these conditions reasonably stable films were formed such that individual voltammograms could be measured over a range of different pH values. From these measurements, it was clear that higher current densities are achieved with *Tm*HydA (up to − 0.9 mA cm^−2^ for H^+^ reduction) (Fig. 3b) than *Tm*HydABC (up to − 0.045 mA cm^−2^ for H^+^ reduction) (Fig. 3a), but otherwise, the two enzymes behaved in a similar fashion showing a bias towards H^+^ reduction at neutral to low pH. The difference in current densities between *Tm*HydA and *Tm*HydABC could be related to the larger size of *Tm*HydABC (160 kDa) compared to *Tm*HydA (72 kDa), which may lead to higher electroactive coverage (surface concentration of the electroactive enzyme) for *Tm*HydA, as more enzymes can fit into the same surface area. The oligomeric nature of the enzyme can also influence the electroactive coverage: *Tm*HydABC forms a tetramer of trimers in solution (around 700 kDa), while *Tm*HydA behaves as a monomer or dimer (Figures S2 and S3). Protein film electrochemistry reveals that for both *Tm*HydABC and *Tm*HydA, the H^+^ reduction current is strongly pH dependent, dominating over H_2_ oxidation at pH values below 8, while the H_2_ oxidation current is relatively pH independent (Fig. 3). Both enzymes show reversible electrochemical behavior with essentially no over-potential requirement in either direction (Fig. 3). The behavior of *Tm*HydA is reminiscent of that from *Clostridium pasteurianum* and *Clostridium acetobutylicum* [FeFe] hydrogenases both of which show a bias towards H^+^ reduction at neutral pH [9, 39].

### Catalytic activity using physiological partners

**Fig. 4:**
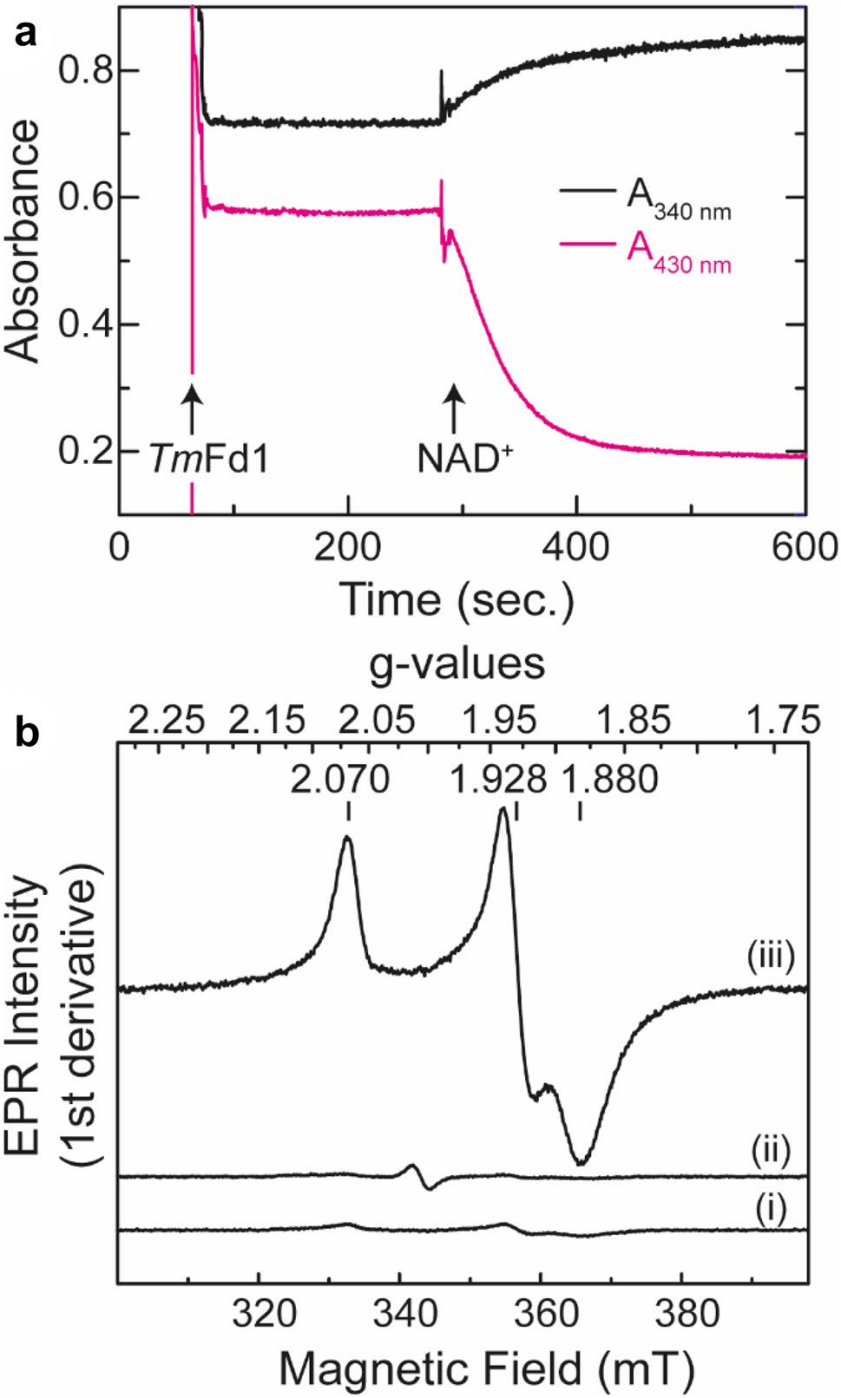
Activity assay of *Tm*HydABC using physiological partners. **a** Simultaneous reduction of *Tm*Fd1 (decrease in *A*_430nm_) and NAD^+^ (increase in *A*_340nm_) by *Tm*HydABC at 70 °C under an atmosphere of 100% H_2_ was monitored by UV–Vis spectroscopy. To a 1 mL reaction mixture containing  ≈ 680 ng *Tm*HydABC and 50 µM FMN in 200 mM potassium phosphate (pH 8) buffer, ≈35 µM *Tm*Fd1 was added (indicated by the first arrow). To the same reaction mixture, 0.5 mM NAD^+^ was added (indicated by the second arrow). **b** Reduction of *Tm*Fd1 monitored by EPR spectroscopy. In the absence of *Tm*HydABC, H_2_ did not reduce *Tm*Fd1 (trace i). In the absence of NAD^+^, *Tm*HydABC could not catalyze reduction of *Tm*Fd1 from H_2_ (trace ii). However, when NAD^+^ is present, *Tm*HydABC could reduce *Tm*Fd1 using H_2_ as indicated by the appearance of the typical rhombic spectrum corresponding to the reduced [4Fe–4S] cluster. The conditions of the reactions used for preparing EPR samples were the same as for the UV–Vis measurements. The EPR spectra were measured at 10 K and at 0.2 mW power

The *Tm*HydABC enzyme isolated from the native organism was unable to produce H_2_ with reduced ferredoxin as the sole electron donor [14]. However, when reduced ferredoxin and NADH were added together as electron donors, *Tm*HydABC could evolve H_2_ [14]. Thus, *Tm*HydABC acts as an electron-confurcating (reverse of bifurcation) hydrogenase, which converges electrons from two different sources to reduce protons to H_2_ [14, 23]. To check if the recombinantly produced and artificially maturated *Tm*HydABC is able to perform electron bifurcation, we followed the reverse reaction, i.e., we monitored H_2_ oxidation coupled to reduction of NAD^+^ and ferredoxin. The ferredoxin (*Tm*Fd1) used for this assay is a single [4Fe–4S] cluster containing ferredoxin from *T. maritima* (details of its purification and characterization are provided in the Supplementary Text 4 and Figure S8). *Tm*HydABC was not able to reduce *Tm*Fd1 in an H_2_-saturated assay mixture containing FMN but lacking NAD^+^ (indicated by the unchanged absorbance intensity at 430 nm) (Fig. 4a). As soon as NAD^+^ was added to the assay, a decrease in the absorbance at 430 nm was observed, indicating reduction of *Tm*Fd1 (Fig. 4a). Concomitantly, NADH production was detected by an increase of the absorbance at 340 nm (Fig. 4a). The changes in the absorbance intensities at 340 nm and 430 nm were not observed in the absence of *Tm*HydABC, indicating that the H_2_-dependent reduction of NAD^+^ and *Tm*Fd1 is indeed catalyzed by *Tm*HydABC.

The above observation was also confirmed by EPR spectroscopy (Fig. 4b). The reaction mixture containing NAD^+^ and *Tm*Fd1, but lacking *Tm*HydABC, does not show EPR signals corresponding to the reduced [4Fe–4S] cluster of *Tm*Fd1 (Fig. 4b, trace i). Similarly, no [4Fe–4S] cluster EPR signal was observed when the reaction was performed without NAD^+^ (Fig. 4b, trace ii). The typical rhombic feature of the reduced [4Fe–4S] cluster originating from *Tm*Fd1 was observed only when the reaction mixture contained both NAD^+^ and catalytic amounts of *Tm*HydABC (Fig. 4b, trace iii). These results indicate that our holo-*Tm*HydABC is capable of electron bifurcation in the presence of its physiological redox partners.

The specific activity of NADH production by *Tm*HydABC in the presence of *Tm*Fd1 at 70 °C was found to be ≈16.6 U mg^−1^ (Table S1), which is well within the range that was obtained for other electron-bifurcating [FeFe] hydrogenases [21, 27–29, 40]. The stoichiometry of NADH produced vs *Tm*Fd1 reduced was found to be 1:2.01 (± 0.1), indicating that the reaction proceeds as expected according to Eq. (1) (Figure S9). Like the electron-bifurcating [FeFe] hydrogenase from *Desulfovibrio fructosovorans* (Hnd) [29], *Tm*HydABC was also able to reduce NAD^+^ in the absence of *Tm*Fd1, however, at ≈35-fold lower rate (Table S1). The specific activity of NADH production was found to be ≈fivefold lower (3.4 ± 0.5 U mg^−1^) when flavin adenine dinucleotide (FAD) was added to the assay buffer instead of FMN (Table S1). A similar observation was also made in the case of natively isolated *Tm*HydABC that showed approximately 50% activity when FAD was added instead of FMN [14]. It is interesting to note that even in the absence of any FMN or FAD artificially maturated *Tm*HydABC could form NADH at ≈fourfold lower rate (4.7 ± 0.6 U mg^−1^) than when excess FMN was added to the assay mixture (Table S1, Figure S10). This residual activity appears to arise from the fraction of *Tm*HydABC molecules that retained the FMN cofactor (0.2 ± 0.05 moles of FMN per mole of *Tm*HydABC). From this observation, it can be concluded that the flavin cofactor required for the optimal bifurcation activity of *Tm*HydABC is FMN and not FAD. Furthermore, this observation supports the idea that only one FMN per HydABC trimer is required for bifurcation and that a second flavin is highly unlikely to be present. Since the addition of excess FMN leads to an ≈fourfold increase in rate, this indicates that the FMN content has likewise increased ≈fourfold (from ≈ 0.2 FMN per HydABC to ≈ 0.8). If there were two sites, excess FMN would be expected to increase the FMN content ≈tenfold, leading to a greater than tenfold increase in the rate. This finding has important implications for the bifurcation mechanism, which are discussed below.

### Spectroscopic characterization of the holo-protein

The CO and CN^−^ ligands coordinated to the iron atoms in the H-cluster have characteristic stretching vibrations in the range 2150–1750 cm^−1^, where the characteristic amide vibrations from the protein backbone are absent. Since the stretching vibrations of these ligands are sensitive to changes in oxidation state of the metal center, Fourier transform infrared (FTIR) spectroscopy is an effective tool to study the redox intermediates of the H-cluster. The characteristic FTIR bands for the various redox states of some well-characterized [FeFe] hydrogenases are presented in Table S2.

EPR spectroscopy is an additional tool providing information about the paramagnetic species present in the various redox states of the hydrogenase. Characteristic *g* values for the redox states of some well-characterized hydrogenases are presented in Table S3.

### Characterization of the oxidized state

**Fig. 5:**
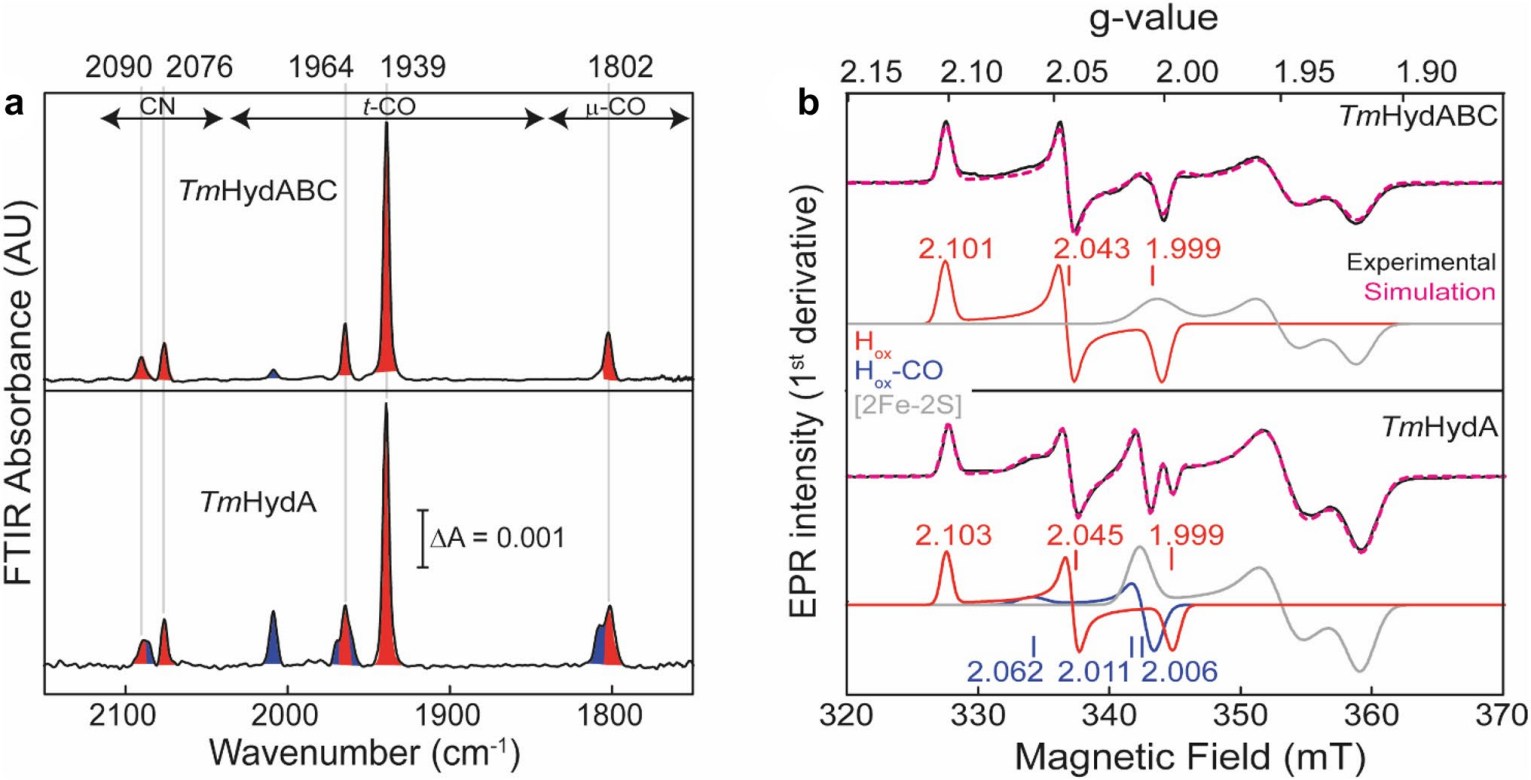
FTIR and EPR spectra of artificially maturated *Tm*HydABC and *Tm*HydA under oxidizing conditions. **a** Top panel shows the FTIR spectrum of 400 µM *Tm*HydABC oxidized with 20 mM NAD^+^ and the lower panel shows the FTIR spectrum of 400 µM *Tm*HydA oxidized with 1 mM thionine. Both samples were in 0.1 M Tris–HCl pH 8 buffer, 0.15 M NaCl and the spectra were measured at room temperature. The peaks belonging to the H_ox_ state are shaded in red and those belonging to the H_ox_–CO state are shaded in blue. **b** CW X-band EPR spectra of the oxidized *Tm*HydABC and *Tm*HydA samples at 20 K, 0.1 mW microwave power. The samples were prepared in the same way as for the FTIR measurements. The experimental spectra are overlaid with spectral simulations (dotted magenta lines) and the component spectra are shown underneath. The red trace (Component 1) corresponds to the H_ox_ state, the blue trace (Component 2) corresponds to the H_ox_–CO state and the gray trace (Component 3) corresponds to one of the reduced F-clusters

After artificial maturation, both *Tm*HydABC and *Tm*HydA were obtained in a mixture of oxidation states (Figure S6). Various redox agents were used to enrich the individual oxidation states. Figure 5 shows the FTIR and EPR spectra of *Tm*HydABC and *Tm*HydA after oxidizing the proteins with NAD^+^ and thionine, respectively. In both cases, the FTIR spectra (Fig. 5a) are characterized by five major FTIR bands (2090, 2076, 1964, 1939, and 1802 cm^−1^). The positions of these bands are comparable to those reported for the H_ox_ state of *Tm*HydABC isolated from the native organism [30] and also to those reported for other [FeFe] hydrogenases (Table S2). It is, therefore, reasonable to assume that the H-cluster of both *Tm*HydABC and *Tm*HydA are mostly in the H_ox_ state under the applied oxidizing conditions.

In the H_ox_ state, both irons in the [2Fe]_H_ subsite are in a low spin configuration leading to an *S* = 1/2 ground state for H_ox_. In this electronic configuration, the H-cluster shows a rhombic EPR spectrum with two *g* values above 2.0. Figure 5b shows the CW X-band EPR spectra of oxidized *Tm*HydABC and *Tm*HydA. The experimental spectra of both *Tm*HydABC and *Tm*HydA were simulated with three components (details of the EPR simulations are given in Table S4). For both *Tm*HydABC and *Tm*HydA, Component 1 is a rhombic EPR spectrum with almost identical *g* values; 2.102, 2.044, and 1.998 for *Tm*HydABC and 2.103, 2.045, and 1.999 for *Tm*HydA (Fig. 5b). These *g* values are in good agreement with the *g* values of the H_ox_ state of other [FeFe] hydrogenases (Table S3). Thus, the H_ox_ state is also observed for *Tm*HydABC and *Tm*HydA by EPR after oxidative treatment. The additional Component 2 can be assigned to the H_ox_–CO state, and indications of its presence were also observed in the FTIR spectra (Fig. 5a, peaks shaded in blue). The third component with *g* values of [2.013, 1.950, 1.917] for *Tm*HydABC and [2.005, 1.949, 1.918] for *Tm*HydA (Table S4) can be assigned to reduced FeS clusters based on the similarity to the spectra of reduced apo-*Tm*HydABC and apo-*Tm*HydA (Figure S5B). When the EPR spectrum of the NAD^+^ oxidized *Tm*HydABC sample was measured at higher temperature (40 K), signals corresponding to Component 3 could still be observed, which indicated that this signal is originating from a slowly relaxing [2Fe–2S] cluster (Figure S11). EPR spin quantitation on the oxidized holo-*Tm*HydABC sample gave a spin content of ≈ 0.6 spins/molecule of protein for the H_ox_ component and ≈ 0.1 spins/molecule of protein for the H_ox_–CO component. Therefore, the total spin content of the H-cluster is approximately 0.7 spins/molecule of protein. In the case of native *Tm*HydABC the spin content originating from the H-cluster was found to be ≈0.1 spin/molecule of protein [26], indicating a larger proportion of intact H-clusters in the *Tm*HydABC sample prepared by our method, which could also explain the higher activity found in solution compared with that reported for the native enzyme [26].

### Characterization of the CO-inhibited state

**Fig. 6:**
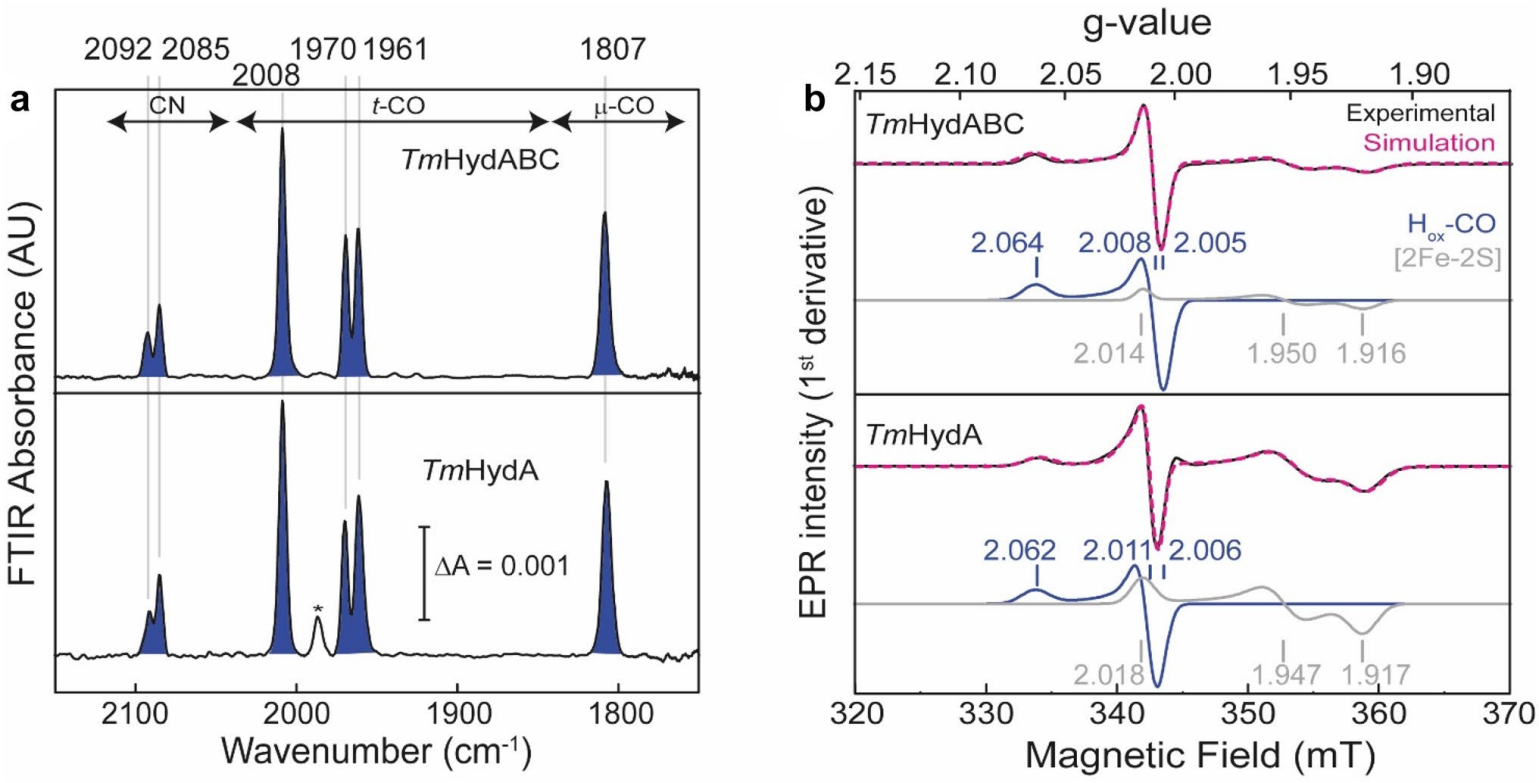
FTIR and EPR spectra of *Tm*HydABC and *Tm*HydA after CO inhibition. **a** FTIR spectra of CO-inhibited (H_ox_–CO) artificially maturated *Tm*HydABC and *Tm*HydA. The ‘as-isolated’ protein samples (≈ 400 µM) were purged for 20 min with 100% CO, followed by incubation for an additional 60 min. Spectra were measured at room temperature. The peak marked with an asterisk belongs to an unidentified species. **b** CW X-band EPR spectra of CO-inhibited (H_ox_–CO) *Tm*HydABC and *Tm*HydA measured at 20 K and 0.1 mW microwave power. Approximately, 150 μM of each protein in 0.1 M Tris–HCl buffer pH 8, 0.15 M NaCl, 20% glycerol, were purged for 20 min with 100% CO, before measuring the spectra. The experimental spectra are overlaid with the spectral simulations (dotted magenta line) and the components are shown underneath

CO is a well-known competitive inhibitor for [FeFe] hydrogenases by occupying the open coordination site of the H-cluster to form the H_ox_–CO state, a well-defined state showing characteristic EPR and FTIR signals [1, 41, 42]. FTIR and EPR spectra of the H_ox_–CO state of *Tm*HydABC and *Tm*HydA are shown in Fig. 6.

FTIR spectra of CO-inhibited *Tm*HydABC and *Tm*HydA are virtually identical and are characterized by the same IR bands at 2092 and 2085 cm^−1^ for the CN^−^ ligands; 2008, 1970 and 1961 cm^−1^ for the terminal CO ligands and 1807 cm^−1^ for the bridging CO ligand. The FTIR bands of the CO ligands are in very good agreement with those recently published for native *Tm*HydABC [30]. Both the CN^−^ and CO IR-stretching frequencies are very similar to those reported for the H_ox_–CO state of other [FeFe] hydrogenases (Table S2).

The experimental EPR spectra of CO-inhibited *Tm*HydABC and *Tm*HydA indicate the presence of two paramagnetic species. The almost axial EPR spectra with *g* values [2.064, 2.008, 2.005] for *Tm*HydABC and [2.062, 2.011, 2.006] for *Tm*HydA are similar to those from the H_ox_–CO state of other [FeFe] hydrogenases (Table S3) and arise from the low spin iron centers in the mixed valence [Fe(I)Fe(II)]_H_, analogous to the H_ox_ state. The oxidized sample of *Tm*HydABC isolated from the native organism showed features at *g* = 2.070, 2.024, and 2.002 [26]. The EPR lines at 2.070 and 2.002 are similar to the H_ox_–CO EPR signature observed here for artificially maturated *Tm*HydABC or for other [FeFe] hydrogenases. The second paramagnetic species observed in the EPR spectra of the H_ox_–CO state can be assigned to a reduced [2Fe–2S] cluster, as it has *g* values and line shapes similar to those observed for the third component in the EPR spectra of the H_ox_ samples.

### Characterization of the reduced state

**Fig. 7:**
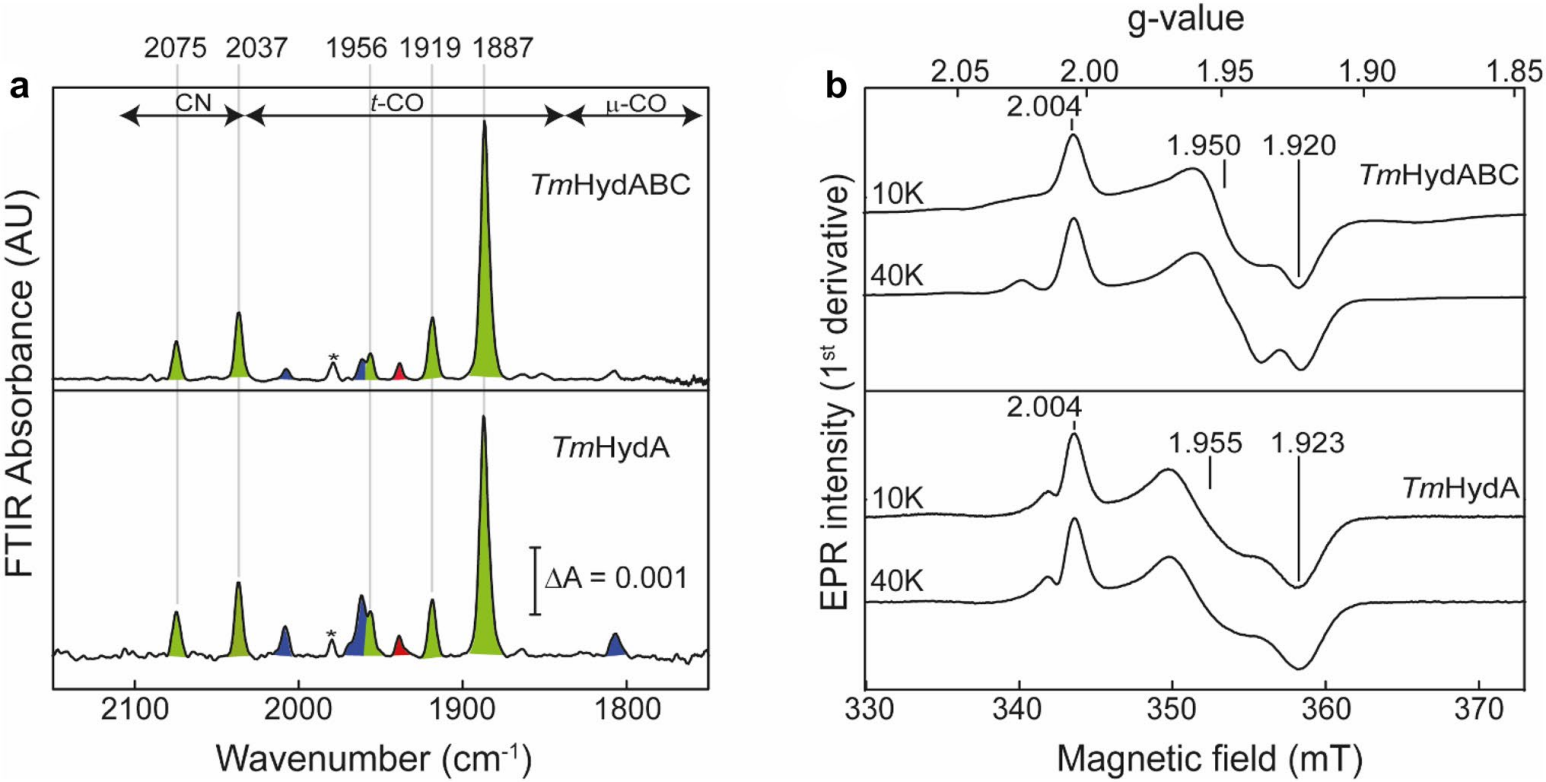
FTIR and EPR spectra of the reduced *Tm*HydABC and *Tm*HydA. **a** FTIR spectra of reduced *Tm*HydABC or *Tm*HydA samples are shown in the upper and lower panels, respectively. Approximately 400 µM of ‘as-isolated’ protein samples were incubated with 20 mM of sodium dithionite (NaDT) at room temperature for 5 min before measuring the spectra at room temperature. The peaks corresponding to the H_red_H^+^ state are shaded in green. The minor peaks shaded in blue and red belong to the H_ox_–CO and H_ox_ states. The peak marked with an asterisk belongs to an unidentified species. **b** CW X-band EPR spectra of reduced protein samples (*Tm*HydABC and *Tm*HydA) were measured at 10 K (0.01 mW microwave power) and 40 K (1 mW microwave power). EPR sample composition: 150 μM, 10 mM of NaDT, 0.1 M Tris–HCl buffer pH 8, 0.15 M NaCl, 20% glycerol

Reduction of the Fe-centers in the H-cluster causes red-shifts of the FTIR peaks (with respect to H_ox_) of the CO and CN^−^ ligands due to increases of electron density in anti-bonding ligand orbitals, which lengthens the CO and CN^−^ bonds [43]. This effect is largest when reduction takes place at the [2Fe]_H_ subcluster (20–50 cm^−1^); however, small red-shifts (≈10 cm^−1^) are also observable when the [4Fe–4S]_H_ subcluster is reduced [8]. When reduced with sodium dithionite, FTIR spectra of *Tm*HydABC and *Tm*HydA are identical and showed five major IR bands at 2075, 2037, 1956, 1919, and 1887 cm^−1^ (Fig. 7a). All these bands are significantly red-shifted (by 20–50 cm^−1^) as compared to the H_ox_ state indicating reduction of the [2Fe]_H_ subsite. They resemble closely those for the H_red_H^+^ state identified in other [FeFe] hydrogenases (see Table S2). It is to be noted here that in the H_red_H^+^ state, the bridging CO vibration is absent in both *Tm*HydABC and *Tm*HydA; however, an additional peak is observed at 1956 cm^−1^ (Fig. 7a), which may indicate that in the H_red_H^+^ state the bridging CO becomes terminal, as was proposed for the H_red_H^+^ state in the [FeFe] hydrogenases from *Chlamydomonas reinhardtii* (*Cr*HydA1) and *Desulfovibrio desulfuricans* (*Dd*HydAB) [8, 44, 45]. However, in a recent study, it was suggested that the bridging CO can be retained in the H_red_H^+^ state under certain conditions [46].

Due to the antiferromagnetic spin coupling in the [Fe(I)Fe(I)] unit, the H-cluster in the H_red_H^+^ state is EPR silent, but the reduced F-clusters give rise to a characteristic EPR spectrum consisting of multiple contributions from [4Fe–4S]^+^ and [2Fe–2S]^+^ clusters (Fig. 7b) very similar to the EPR spectrum observed for the reduced apo-enzyme (Figure S5B) and that of the reduced native enzyme [26, 38, 47]. Interestingly, the dominant contribution with *g* values ([2.004, 1.950, 1.920] for *Tm*HydABC and [2.004, 1.955, 1.923] for *Tm*HydA) is also present in the H_ox_ and H_ox_–CO states (Figs. 5b, 6b) and was tentatively assigned to one of the two [2Fe–2S]^+^ clusters in the HydA subunit.

### Spectroelectrochemical characterization of *Tm*HydABC

**Fig. 8:**
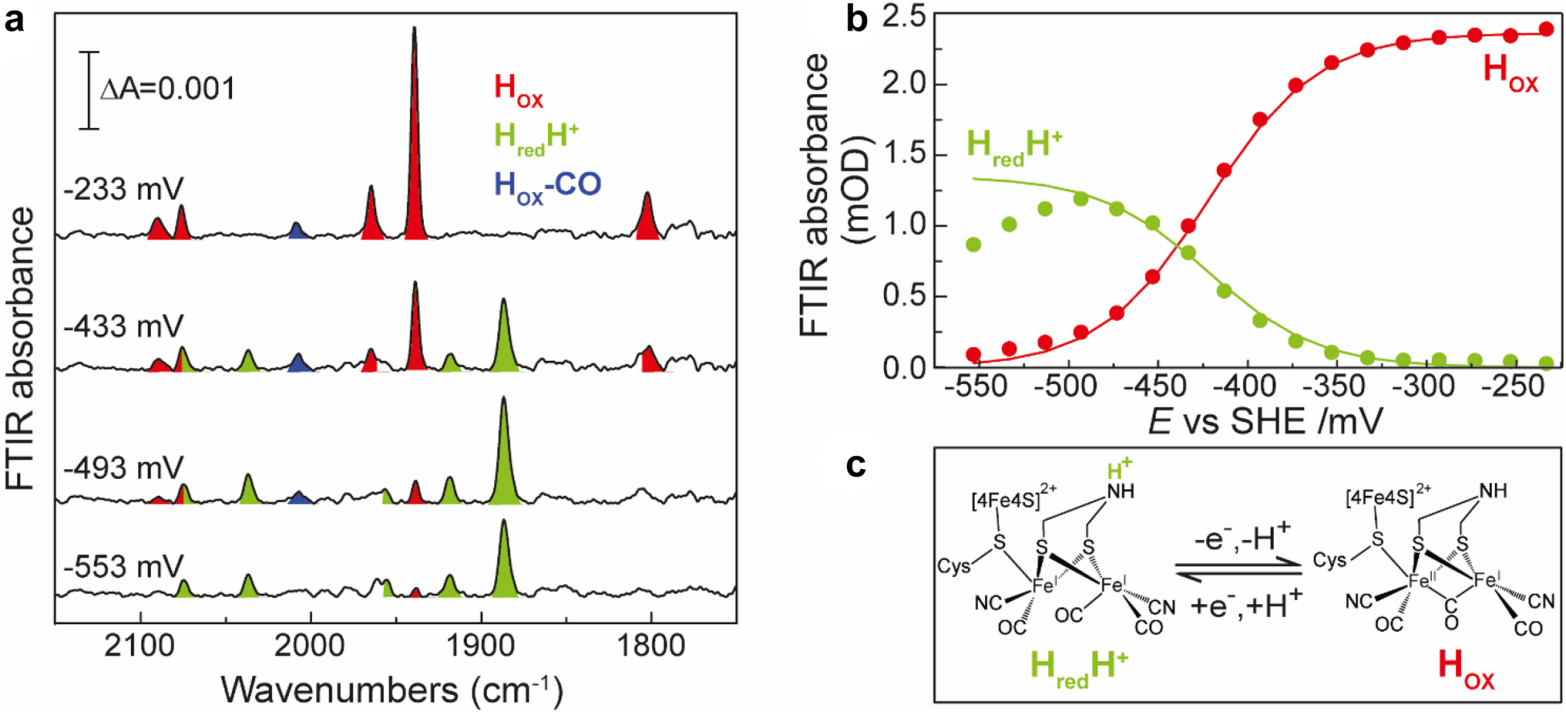
Spectroelectrochemical FTIR of *Tm*HydABC. **a** Selected FTIR spectra recorded at − 233, − 433, − 493 and − 553 mV are shown. The experiment was performed with ≈1 mM *Tm*HydABC in 200 mM phosphate buffer (pH 8) and 200 mM KCl with redox mediators at 15 °C. **b** Changes in FTIR absorbance with changing electrode potential, during reductive titration, at peak position 1939 cm^−1^ (H_ox_) and 1887 cm^−1^ (H_red_H^+^) are shown by red and green circles, respectively. The solid red and green lines represent the Nernst-fit corresponding to the model shown in **c**

Spectroelectrochemical FTIR was used previously in studies of several [FeFe] hydrogenases to investigate their equilibrium redox properties and to calculate midpoint potentials [7, 44]. Here, we apply this method to characterize the potential dependence of the observed redox states in *Tm*HydABC. The reductive titration of *Tm*HydABC was initiated at an open-circuit potential (OCP) of ≈ − 230 mV, vs SHE. The FTIR spectrum recorded at this potential suggested that the protein is mainly in the H_ox_ state (Fig. 8a). As the potential of the cell was decreased, the peaks corresponding to the H_ox_ state decreased in intensity and were replaced by the peaks that were previously observed in the dithionite reduced enzyme (Fig. 8a). However, when the potential was decreased lower than ≈−500 mV, the intensity of these peaks decreased but no new peaks appeared, indicating irreversible degradation of the H-cluster (Fig. 8a, b).

The reductive titration curve obtained by plotting the intensities of the bands at 1939 cm^−1^ (H_ox_) and 1887 cm^−1^ (H_red_H^+^) vs. the applied potential could be fitted using the Nernst equation corresponding to a one-electron reduction (Fig. 8b, c). This fitting revealed that the state characterized by bands at 2075, 2037, 1956, 1919, and 1886 cm^−1^ is indeed one electron reduced with respect to the H_ox_ state, and further supporting that this state is H_red_H^+^. The redox potential associated with the H_ox_/H_red_H^+^ transition for *Tm*HydABC at pH 8 (Fig. 8b) was found to be − 420 ± 5 mV, which is similar to that reported for *Dd*HydAB [44, 45].

The H_red_H^+^ state is formed by proton coupled electron transfer (PCET) of the H_ox_ state [8]. In *Cr*HydA1, which lacks the F-clusters, both H_red_ and H_red_H^+^ can be observed at neutral pH and the apparent pKa of the ADT amine is around 7.2 [8]. In contrast, for *Dd*HydAB which harbors two F-clusters, the apparent pKa was found to be 9.0 [45]. This difference is explained by the influence of the proximal F-cluster on the redox potential of the [4Fe–4S]_H_ subcluster. Redox anti-cooperativity (redox interaction between the F- and H-clusters that decreases the reduction potential of the H-cluster when the proximal F-cluster is reduced and vice versa), disfavors reduction of the [4Fe–4S]_H_ subcluster, thus promoting direct PCET from the proximal F-cluster to the [2Fe]_H_ subcluster upon reduction of the H-cluster. This mechanism increases the apparent pKa of the ADT amine moiety [45]. During the spectroelectrochemical titration of *Tm*HydABC at pH 8, we did not observe any H_red_ state (Fig. 8). The H_red_ state was also not observed when the FTIR spectrum of dithionite reduced *Tm*HydABC was measured at pH 10 (Figure S12). The exceptionally high apparent pKa of the ADT amine in *Tm*HydABC could be the result of structural modifications in the H-cluster binding pocket with respect to *Dd*HydAB and *Cr*HydA1 that reinforce the effect of redox anti-cooperativity with the proximal F-cluster(s).

Artificial maturation with the unprotonatable [2Fe]^ADT^-analogue [2Fe]^PDT^ [48, 49] confirmed that an unprotonated reduced state of *Tm*HydABC is indeed accessible. The [2Fe]^PDT^-maturated *Tm*HydABC exhibits an H_red_ state with very low signal intensity and broadened peaks (Figure S13). The reason for this is unclear, but may be related to some conformational freedom of the [2Fe] site in this enzyme. Furthermore, we found no evidence for the formation of a two electron reduced state (the H_sred_H^+^ state) in our spectroelectrochemistry data (Fig. 8). Formation of the H_sred_H^+^ state was proposed to be essential for *Cr*HydA1 during H_2_ production [9]. For *Dd*HydAB, an [FeFe] hydrogenase containing F-clusters, it was proposed that it can avoid the formation of the H_sred_H^+^ state, as the nearby F-clusters can accept the second electron and can tunnel it quickly to the H-cluster during catalysis [45]. A similar phenomenon is also likely to be operative in *Tm*HydABC leading to the absence of H_sred_H^+^, although the protein is catalytically highly active. It should be noted that in a recent kinetics study on native *Tm*HydABC, the bands assigned to H_red_H^+^ in our study were attributed to the doubly reduced state H_sred_H^+^ with some contribution from H_red_H^+^ (referred to as “H_sred_” and “H_red_”, respectively) [30]. We believe that as the positions of the FTIR bands of the H_red_H^+^ and H_sred_H^+^ states are very similar, correct assignment of bands to these two states is difficult without electrochemical titrations. The main conclusions of this kinetics study, however, are not affected by this misassignment [30]. The protonated reduced state did occur under proton reduction conditions and was identified as a catalytically active state [30].

## Discussion

In this study, the heterotrimeric electron-bifurcating [FeFe] hydrogenase from *T. maritima*, *Tm*HydABC, and its catalytic subunit *Tm*HydA was produced by recombinant expression and artificial maturation. With artificial redox partners, semisynthetically produced *Tm*HydABC and *Tm*HydA showed significantly higher H_2_ production (550 ± 50 U mg^−1^ and 475 ± 60 U mg^−1^) and H_2_ oxidation activities (1300 ± 140 and 2000 ± 200 U mg^−1^) at 70 °C compared to the enzyme isolated from the native organism [26]. Native *Tm*HydABC was previously found to use both reduced ferredoxin and NADH as electron donors for H_2_ production, thereby acting as an electron-confurcating hydrogenase [14]. Here, we show that the semisynthetically produced holo-*Tm*HydABC could catalyze the reverse reaction, i.e., H_2_-dependent reduction of ferredoxin only in the presence of NAD^+^, demonstrating that the enzyme is also capable of the electron bifurcation reaction.

We have used FTIR and EPR spectroscopy to analyze the salient features of the H-cluster in *Tm*HydABC and *Tm*HydA under various conditions. Under oxidizing conditions, both *Tm*HydABC and *Tm*HydA showed spectral properties (positions of the FTIR peaks and EPR *g* values) similar to the H_ox_ state of prototypical [FeFe] hydrogenases (non-electron bifurcating) (Fig. 5, Tables S1 and S2). Furthermore, when we treated *Tm*HydABC and *Tm*HydA with CO, FTIR, and EPR spectroscopic features of the H_ox_–CO state could be identified. This observation implies that like prototypical [FeFe] hydrogenases, the H-cluster of electron-bifurcating hydrogenases is also inhibited by CO. All these results suggest that properties of the H-cluster of *Tm*HydABC are similar to those of the prototypical [FeFe] hydrogenases. This was expected as the amino acid sequences surrounding the H-cluster pocket are well conserved in electron-bifurcating and non-electron-bifurcating [FeFe] hydrogenases [50]. However, the previously published EPR spectroscopic analysis of native *Tm*HydABC did not identify the characteristic EPR signal of the H_ox_ state [26], which led to the speculation that the H-cluster of electron-bifurcating enzymes is different from prototypical enzymes [51].

Electron bifurcation refers to the process of splitting the electrons from a single electron donor to two different electron acceptors, one with higher redox potential and the other with lower redox potential than that of the electron donor [16, 17]. Electron-bifurcating enzymes usually contain at least one flavin along with several other redox centers and it is presumed that the bifurcation reaction happens at this flavin as this cofactor is capable of both one and two electron transfer reactions (flavin-based electron bifurcation, FBEB) [16, 51]. In general, the flavin centers responsible for FBEB show special redox properties [52]. These flavins display ‘crossed’ redox potentials: they undergo 2e^−^ reduction from the flavoquinone state to the flavohydroquinone state and, then, upon 1e^−^ oxidation, form a highly reducing flavosemiquinone state that can transfer electrons to low potential electron acceptors.

FBEB has been suggested to operate in *Tm*HydABC and other electron-bifurcating [FeFe] hydrogenases [23]. Protein sequence analysis of *Tm*HydABC and other electron-bifurcating [FeFe] hydrogenases indicates the presence of one FMN-binding site in HydB. Based on the sequence similarity between HydB and the NuoF (Nqo1) subunit of respiratory complex I [23], whose FMN does not engage in electron bifurcation, it seems unlikely that the FMN in HydB would be the site of electron bifurcation. Thus, the involvement of a second flavin for electron bifurcation was proposed in *Tm*HydABC [23]. Here, we showed that *Tm*HydABC containing 0.2 FMN per heterotrimer catalyzed the electron bifurcation reaction with a rate of 28% of that in the presence of added excess FMN. This fits with the idea that only one FMN per trimer is necessary for electron bifurcation. If two FMN sites were present then the 0.2 FMN would be split between the two sites, with 0.1 FMN per site (or 10% occupancy of each site) assuming equal affinity for both sites. By the law of probabilities then, only 1% of HydABC would have both sites occupied. If both FMN sites were essential for the catalytic mechanism (one for NADH oxidation and one for electron bifurcation), then, in this scenario, the addition of excess FMN should fill up all empty FMN sites and increase the activity accordingly by about 100-fold. Therefore, the modest fourfold increase in activity, contradicts the proposition of a second flavin being the bifurcation center in *Tm*HydABC.

More recently, an alternative model of the bifurcation mechanism in [FeFe] hydrogenases has been postulated by Peters et al. where the H-cluster was hypothesized to be the electron bifurcation site [51]. The rationale behind this hypothesis was that the H-cluster fulfills one of the key requirements of being an electron-bifurcation center, as it is capable of 2e^−^ redox reactions and can exist in different oxidation states. Like electron-bifurcating flavins, the intermediate redox state of the H-cluster should be strongly reducing in order for it to be an electron bifurcation center [52]. Our spectroelectrochemical analysis of *Tm*HydABC shows that the H-cluster forms a stable 1e^−^ reduced state (H_red_H^+^). The 2e^−^ reduced state (H_sred_H^+^) was not observed under our experimental conditions; possibly due to a very low redox potential of the H_red_H^+^ ⇆ H_sred_H^+^ transition imposed by redox anti-cooperativity between the H-cluster and the reduced F-clusters [45]. This observation implies that the H-cluster does not show ‘crossed-over’ redox behavior similar to electron-bifurcating flavins. Although according to Zhang et al. redox centers with uncrossed redox potentials can also theoretically act as bifurcating centers under certain conditions [55].

A possible mechanism would be that H_2_ oxidation at the H-cluster produced a highly reducing H_sred_H^+^ species, which quickly transfers an electron to a nearby iron–sulfur cluster with a very negative redox potential. This would then be followed by downhill electron transfer to the ferredoxin-binding site. The H_red_H^+^ state would then transfer an electron to a different iron–sulfur cluster with a more positive redox potential. We think that this is rather unlikely to be the case in *Tm*HydABC, due to the high sequence similarity between *Tm*HydA and the structurally characterized [FeFe] hydrogenase from *C. pasteurianum* (*Cp*I or *Cp*HydA) [4, 54]. *Cp*HydA contains four accessory iron–sulfur clusters, but only one of them is within electron transfer distance of the H-cluster. Thus, a second proximal iron–sulfur cluster in *Tm*HydA seems unlikely.

**Fig. 9:**
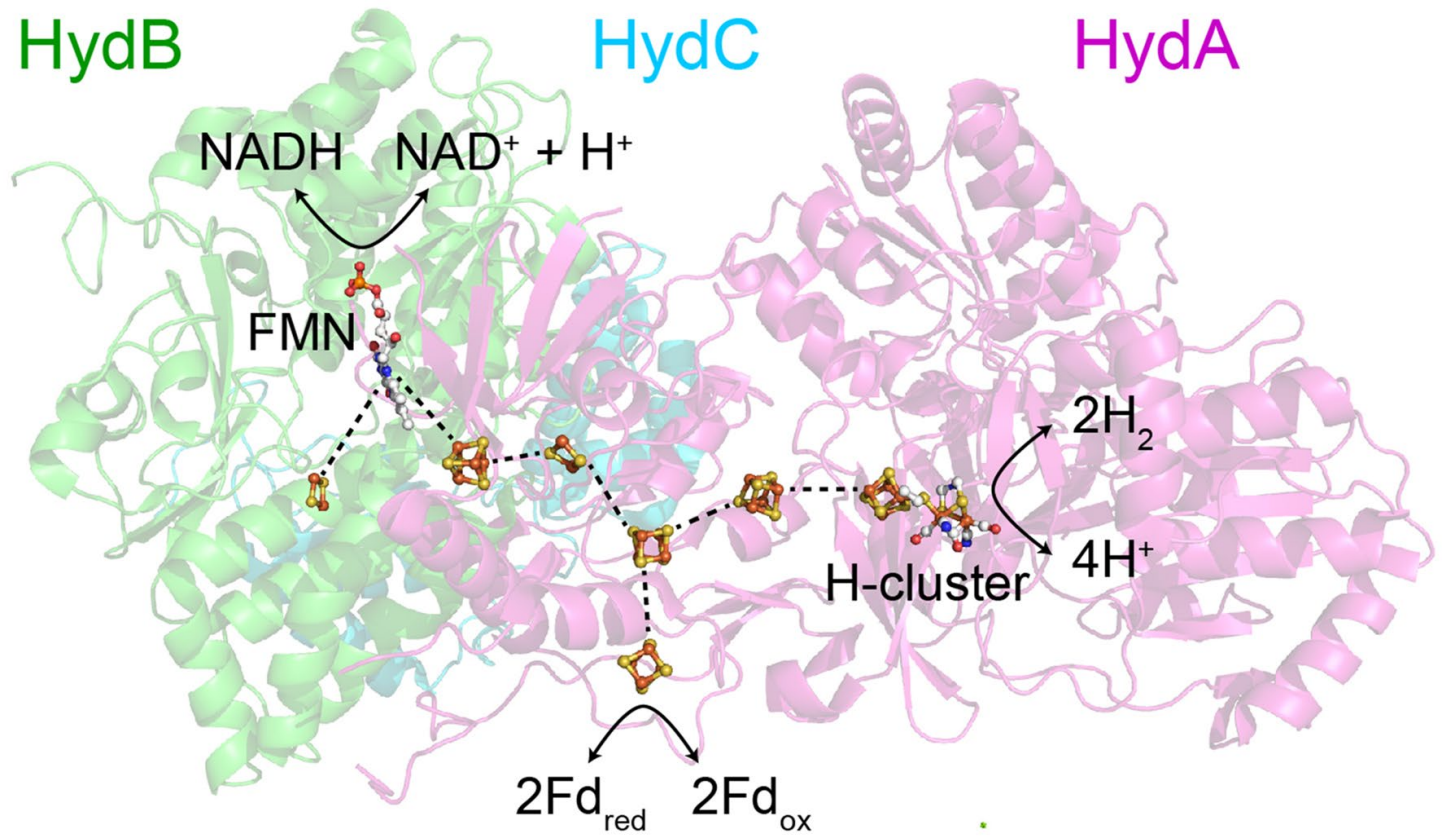
Proposed arrangement of subunits and cofactors in *Tm*HydABC. The *Tm*HydABC subunits are homologous to the Nqo1, Nqo2, and Nqo3 subunits of the structurally characterized complex I from *Thermus thermophilus*. Based on this homology, the arrangements of the conserved cofactors can be predicted. The figure shows the protein subunits Nqo1 (HydB, green), Nqo2 (HydC, blue), and Nqo3 (HydA, pink) in the cartoon representation (PDB: 4HEA [53],), with the cofactors from Nqo1 and Nqo2 from complex I, and the cofactors from the [FeFe] hydrogenase *Cp*HydA (PDB: 4XDC [54]), overlaid. *Cp*HydA was aligned to Nqo3 in Pymol giving almost perfect alignment of the homologous clusters. HydA contains an additional [2Fe–2S] cluster, for which *Cp*HydA does not contain a homologous cluster, and HydB contains an additional two [4Fe–4S] clusters and one [2Fe–2S] cluster, for which complex I does not contain homologous clusters

We hypothesize, instead, that a complicated arrangement of iron–sulfur clusters, and interactions between them may facilitate a novel elegant electron-bifurcating mechanism. The arrangement of cofactors and subunits in *Tm*HydABC remains unknown. However, the strong homology between *Tm*HydA, *Tm*HydB, and *Tm*HydC, and the complex I subunits *Tt*Nqo1, *Tt*Nqo2, and *Tt*Nqo3 from *Thermus thermophilus* [53], may indicate a similar arrangement of subunits and cofactors (Fig. 9). In this arrangement, HydC would be located on one side of HydB, positioning the [2Fe–2S] cluster of HydC close to the FMN cofactor of HydB. Meanwhile, HydA would be located on the opposite side of HydB with the surface exposed [2Fe–2S] cluster of HydA in electrical contact with the surface exposed [4Fe–4S] cluster of HydB. This arrangement would not be compatible with the previously proposed Fd-binding site being HydC. Instead, Fd would interact with the His-ligated [4Fe–4S] cluster of HydA, as has been proposed for the *Cp*HydA [56]. With the H-cluster in one direction, the FMN in another direction and the Fd-binding site in a third direction, this arrangement temptingly implicates the trinity of iron–sulfur clusters in HydA as a potential bifurcation site. How, such an arrangement could operate to regulate electron transfer from the H-cluster in one direction or another is unclear, but we speculate that the His-ligated [4Fe–4S] cluster in HydA could play an important role. Further investigations are underway to investigate this possibility. Crucially, our recombinant method for producing *Tm*HydABC represents the perfect system to perform such in-depth mechanistic studies of the electron-bifurcating mechanism, since it provides an efficient way to produce very high yields of pure protein, as well as making it easy to produce site directed mutations to directly test these ideas.

## Conclusion

In this study, we have developed a method of producing the heterotrimeric electron-bifurcating [FeFe] hydrogenase from *T. maritima* using recombinant expression and artificial maturation. The time efficiency of the recombinant expression method prevented protein damage and led to high catalytic activity for both *Tm*HydABC and *Tm*HydA, outperforming enzymes isolated from the native organism. Our preparation was competent in the electron bifurcation reaction, even in the absence of added FMN. Using FTIR and EPR spectroscopy the three typical states present in all active [FeFe] hydrogenases, i.e., H_ox_, H_red_H^+^, and H_ox_–CO could be identified in both *Tm*HydABC and *Tm*HydA. The unprotonated singly reduced state H_red_ as well as the doubly reduced state H_sred_H^+^ (both with a reduced [4Fe–4S]-subcluster) were not observed under any condition. This is taken as evidence for a strong electronic coupling between the H-cluster and the F-clusters in the enzyme disfavoring reduction of the cubane subcluster. Our results do not agree with the FMN or the H-cluster as being the site of electron bifurcation. Instead, we hypothesize that the iron–sulfur clusters in the HydA subunit could serve this function. The efficient method presented here for obtaining high quantities of high quality *Tm*HydABC should pave the road for more protein intensive experiments such as X-ray crystallography, spectroelectrochemistry, and nuclear resonance vibrational spectroscopy, to test our hypothesis, and to help understand the enigmatic mechanism of electron bifurcation in this fascinating [FeFe] hydrogenase.

## Electronic supplementary material

Below is the link to the electronic supplementary material.

Supplementary material 1 (PDF 1521 kb)

## Abbreviations

NADH: Dihydronicotinamide adenine dinucleotide
NAD^+^: Nicotinamide adenine dinucleotide
Fd_ox_: Oxidized ferredoxin
Fd_red_: Reduced ferredoxin
ADT: 2-azapropane 1,3-dithiolate
FMN: Flavin mononucleotide
FTIR: Fourier transform infrared
EPR: Electron paramagnetic resonance
FAD: Flavin adenine dinucleotide
CW: Continuous wave
OCP: Open-circuit potential
PCET: Proton-coupled electron transfer
FBEB: Flavin-based electron bifurcation
