# Supplementary material for: Spectroscopic and biochemical insight into an electron-bifurcating [FeFe] hydrogenase
Source: J Biol Inorg Chem. 2019 Dec 10;25(1):135–49. doi: 10.1007/s00775-019-01747-1 (PMC7064455; doi:10.1007/s00775-019-01747-1)
Supplement: Supplementary file 1 — Supplementary material 1 (PDF 1521 kb) [file 775_2019_1747_MOESM1_ESM.pdf]

Supplementary information

Semisynthetic production of a fully functional electron bifurcating [FeFe] hydrogenase

Nipa Chongdar, [‡a] Krzysztof Pawlak, [‡a] Olaf Rüdiger,[a] Edward J. Reijerse,[a] Patricia Rodríguez-Maciá,[a] Wolfgang Lubitz,[a] James A. Birrell,*[a] and Hideaki Ogata*[ab]

[a] Max Planck Institute for Chemical Energy Conversion, Stiftstrasse 34-36, 45470 Mülheim an der Ruhr, Germany.
[b] Institute of Low Temperature Science, Hokkaido University, Kita19 Nishi8, Kita-ku, 060-0819 Sapporo, Japan.

Table of Contents Page

Supplementary text 1: Cloning and heterologous expression of *Tm*HydABC and *Tm*HydA	S2
Supplementary text 2: Purification and characterization of the apo-*Tm*HydABC and *Tm*HydA	S3
Supplementary text 3: Artificial maturation	S3
Supplementary text 4: Purification of *Tm*Fd1	S4
Table S1: Specific activities of H_2 dependent reduction of NAD^+ and *Tm*Fd1 under various conditions	S5
Table S2: Collection of FTIR bands of various [FeFe] hydrogenases	S6
Table S3: Collection of g-values of various [FeFe] hydrogenases	S7
Table S4: Collection of EPR simulation parameters	S8
Figure S1: SDS PAGE analysis of purified apo-proteins	S9
Figure S2: Native PAGE analysis of purified apo-proteins	S10
Figure S3: Analytical size exclusion chromatography	S11
Figure S4: MALDI-TOF analysis of apo-*Tm*HydABC	S12
Figure S5: Spectroscopic characterization of apo-*Tm*HydABC and apo-*Tm*HydA	S13
Figure S6: FTIR spectra of *Tm*HydABC and *Tm*HydA after artificial maturation	S14
Figure S7: Sequence alignment of all five ferredoxins found in the genome of *T. maritima*	S15
Figure S8: Characterization of *Tm*Fd1 isolated using recombinant methods	S16
Figure S9: Stoichiometry of NAD^+ and *Tm*Fd1 reduction during electron bifurcation reaction with *Tm*HydABC	S17
Figure S10: Reduction of NAD^+ and *Tm*Fd1 by *Tm*HydABC in the absence of added FMN	S18
Figure S11: CW X-band EPR spectra of oxidized holo-*Tm*HydABC at various temperatures	S19
Figure S12: FTIR spectra of *Tm*HydABC at pH 8 and 10	S20
Figure S13: FTIR spectra of *Tm*HydABC activated with $[2Fe]^{PDT}$	S21
References	S22

Supplementary text 1

Cloning and heterologous expression of *Tm*HydABC and *Tm*HydA

The genes encoding *Tm*HydA, *Tm*HydB and *Tm*HydC subunits (TM1424-TM1426) were amplified by PCR from *Thermotoga maritima* MSB8 genomic DNA (ATCC, 43589D-2) using the following primers: 5′-TATACCGCGGTGTTGGCGTTGGAAAGACAC-3′ (forward) and 5′-TGTACTGCAGTCAGCCATTTTTCGAAAGCTCC-3′ (reverse). The amplified genes were then ligated between the SacII and PstI sites of the pASK-IBA17plus vector (IBA). During expression the pASK(*strephydcba*) construct would yield the *Tm*HydABC protein with a StrepII-tag and TEV-protease cleavage sequence at the N-terminus of the *Tm*HydC subunit. A pASK(*strephyda*) construct was generated from the pASK(*strephydcba*) construct using PCR with the primers 5′-TATAGGCGCCATGAAAATTTACGTTGATGG-3′ (forward) and 5′-TATAGGCGCCCTGAAAATAAAGATTC-3′ (reverse), followed by digestion with KasI (NEB) and circularization with T4 DNA ligase (NEB). The strep-tag and TEV-site were removed from pASK(*strephyda*) by PCR using the primers 5′-ATGAAAATTTACGTTGATGGAAG-3′ (forward) and 5′-TTG TATATCTCCTTCTTAAAGTTAAAC -3′ (reverse) followed by blunt-end ligation giving pASK(*hyda*). A C-terminal strep-tag sequence was added to pASK(*hyda*) using the primers 5′-CCGCAGTTCGAAAAATGACTGCAGGGGGACC-3′ (forward) and 5′-GTGGCTCCAGCTAGCGCCATTTTTCGAAAGCTCC-3′ (reverse) followed by blunt-end ligation to give pASK(*hydastrep*).

The accuracy of the plasmids was determined by DNA sequencing. The constructs were used to transform competent *E. coli* BL21(DE3)-ΔiscR cells.[1] Transformants were grown aerobically at 37 °C in Terrific Broth (TB) medium [2] supplemented with ampicillin (100 μg/ml), kanamycin (50 μg/ml), 2 mM ferric ammonium citrate, and 3 mM L-cysteine until OD_{600} reached ≈0.5. The cultures were supplemented with 1 mM L-cysteine before induction of protein expression with 200 μg/L anhydrotetracycline. Subsequently, the cultures were transferred to glass bottles, sealed with lids containing PTFE/silicone septa and purged with argon gas for 2 h. Protein expression was carried out for 22 - 24 h at room temperature before harvesting the cultures anaerobically.

Supplementary text 2

Purification and characterization of apo-*Tm*HydABC and apo-*Tm*HydA

All protein purification steps were carried out in an anaerobic chamber (Coy, USA) containing 1.5–2% H_2. The cells were resuspended in buffer (100 mM Tris-HCl pH 8.0, 150 mM NaCl, 0.2 mM Pefabloc SC (Sigma)), sonicated for 6 cycles of 1 min (50% duty, 50% power) with 2 min waiting in between, and clarified by centrifugation for 30 min at 18000 x g. The supernatant was filtered (0.44 µm filter (Millipore)) and loaded on a Streptactin column (IBA) pre-equilibrated in buffer (100 mM Tris-HCl, pH 8, 150 mM NaCl). The column was washed with the same buffer before eluting the protein with buffer containing 2.5 mM D-desthiobiotin. The eluted proteins were concentrated using centrifugal concentrators (50 kDa MWCO for *Tm*HydABC and 30 kDa MWCO for *Tm*HydA, Millipore). Some excess HydC was present in the purified *Tm*HydABC (Figure S1 – lane 1). *Tm*HydABC was separated from excess *Tm*HydC by gel-filtration (Superdex 200) using the same buffer (100 mM Tris-HCl, pH 8 and 150 mM NaCl) (Figure S1 – lane 2). Finally, the protein was concentrated to ≈0.5 mM and stored at -80 °C until further use. Protein concentrations were routinely measured using the Biorad DC protein assay. N-terminal-strep-tagged apo-*Tm*HydA did not express well (data not shown) and so the strep-tag was moved to the C-terminus. The C-terminal-strep-tagged protein, in contrast, gave good levels of expression (Figure S1 - lane 3). Similar observations were made with the [FeFe] hydrogenases from *Clostridium pasteurianum* (*Cp*HydA1) and *Desulfovibrio desulfuricans* (*Dd*HydAB), and HydS from *T. maritima*.[1, 3, 4]

MALDI TOF-MS (Voyager DE Pro, Applied Biosystems) was used to determine the precise molecular masses of each subunit of *Tm*HydABC. MALDI TOF-MS confirmed the molecular masses for each of the subunits: strep-tagged *Tm*HydC (21202 m/z, calculated 21251, 192 a.a.), *Tm*HydB (68909 m/z, calculated 68681, 626 a.a.), and *Tm*HydA (72619 m/z, calculated 72254, 645 a.a.) (Figure S4).

The oligomeric nature of the proteins in solution was determined by native-PAGE electrophoresis and analytical gel-filtration chromatography. Native PAGE analysis of purified apo-*Tm*HydABC (Figure S2 – lane 1) and apo-*Tm*HydA (Figure S2 – lane 2) showed that the apo-*Tm*HydABC exists primarily as a tetramer of trimers with a total molecular weight ≈700 kDa (the expected molecular mass of the apo-*Tm*HydABC trimer is ≈ 160 kDa) and apo-*Tm*HydA exists primarily as a monomer or dimer. Analytical size exclusion chromatography gave molecular weights of ≈630 kDa for apo-*Tm*HydABC and ≈ 90 kDa for apo-*Tm*HydA in agreement with native PAGE analysis (Figure S3). Similar oligomeric properties were observed for native *Tm*HydABC.[5] The flavin and iron content of the samples were measured using methods described previously.[6, 7]

Supplementary text 3

Artificial maturation

Artificial maturation of apo-*Tm*HydABC and apo-*Tm*HydA was performed using the method described by Esselborn *et al.*[8] $(Et_4N)_2[Fe_2(adt)(CO)_4(CN)_2]$ was synthesized as described previously.[9] The apo-protein (≈0.4 mM) was mixed with a 2-fold excess of $(Et_4N)_2[Fe_2(ADT)(CO)_4(CN)_2]$ (from a 50 mM stock prepared in anhydrous DMSO) in 100 mM Tris-HCl pH 8.0, 150 mM NaCl buffer and incubated for an hour at room temperature under anaerobic conditions. The protein was then purified from excess $(Et_4N)_2[Fe_2(ADT)(CO)_4(CN)_2]$ using a PD-10 desalting column (GE Lifesciences) pre-equilibrated with 100 mM Tris-HCl, 150 mM NaCl, pH 8 buffer. After this procedure, the hydrogenases were found to be in a state resembling the CO-inhibited state as in other [FeFe] hydrogenases (Figure S6). The samples were shaken under a 2% H_2/ 98% N_2 atmosphere until most of the CO was removed (Figure S6) before it was concentrated to ≈1 mM and stored at -80 °C. It is noteworthy, that the samples prepared by artificial maturation (after overnight shaking) contain a lower percentage of the CO-inhibited state than the as-isolated protein samples obtained from the native organism.[10]

Supplementary text 4

Purification of *T. maritima* ferredoxin

The *T. maritima* genome contains five ferredoxin genes (Fig. S7). Among them, *Tmari*0929 (*Tm*Fd1) the single [4Fe-4S] cluster containing ferredoxin is most well characterized and was used by Schut and Adams to demonstrate the bifurcation activity of *Tm*HydABC isolated from the native organism.[11] To perform the bifurcation assay of recombinantly produced *Tm*HydABC, the codon optimized gene of *Tm*Fd1 with N-terminal Strep-tag II (followed by a TEV protease site) was ordered from GenScript. This gene was inserted in between the NdeI and HindIII sites of the pET21b vector.

E.coli BL21(DE3) ΔiscR competent cells were transformed with the plasmid containing the *Tm*Fd1 gene. Transformants were grown in LB media (buffered with phosphate) supplemented with 100 µg/mL ampicillin, 50 µg/mL kanamycin, 2 mM ferric ammonium citrate, and 1 mM L-cysteine until the OD_{600} reached ≈ 0.5. The cultures were then transferred into glass bottles, sealed with lids containing PTFE/silicone septa and supplemented with 1 mM cysteine and 0.5 mM IPTG to induce protein overexpression. The cultures were then purged with argon for 2 h and left overnight at room temperature to continue with the overexpression of the protein. The cells were then harvested under an anaerobic atmosphere (1.5–2% H_2). All the purification steps were carried out in the anaerobic chamber. The harvested cells were resuspended in buffer (100 mM Tris-HCl pH 8.0, 150 mM NaCl, 0.2 mM Pefabloc SC (Sigma)), lysed by sonication (4 cycles of 3 min (50% duty, 20% power)) and the cell lysate was clarified by centrifugation for 1 h at 18000 x g. The supernatant was filtered through a 0.44 µm filter before loading onto a pre-equilibrated Streptactin column (IBA). The protein was eluted using 100 mM Tris-HCl pH 8.0, 150 mM NaCl buffer containing 2.5 mM D-desthiobiotin. In order to remove the strep-tag II, the eluted protein was digested overnight at room temperature with TEV protease (in 30:1 ratio). The digested protein was then passed through a Talon^{TM} column to remove the TEV protease and the eluted protein was concentrated using a 3 kDa MWCO centrifugal concentrator (Millipore). The concentration of the protein was measured using a UV-vis spectrometer and the protein was stored at -80 ºC.

The purified ferredoxin (*Tm*Fd1) had the expected molecular mass of approximately 7 kDa, and showed similar UV-vis and EPR spectroscopic properties as observed for the native ferredoxin (Fig. S8B and S8C).[12] Using cyclic voltammetry, the midpoint potential of the cluster of *Tm*Fd1 was found to be -410 mV, which agrees with the value reported in the literature (Fig. S8D).[13]

Table S1: Specific activities of H_2 dependent reduction of NAD^+ and *Tm*Fd1 catalyzed by holo *Tm*HydABC measured under various conditions

Condition	Specific activity (U mg^{-1})	
	$NAD^+ \rightarrow NADH$	$Fd_{ox} \rightarrow Fd_{red}$
Without *Tm*HydABC	<0.1	<0.1
0 mM NAD^+	N.A.	<0.1
0 µM *Tm*Fd1	0.5 ± 0.1	N.A.
0 µM FMN	4.7 ± 0.6	9.0 ± 1.7
50 µM FMN	16.6 ± 0.2	33.5 ± 2
50 µM FAD	3.4 ± 0.5	8.2 ± 0.2

1U = 1 µmol min^{-1}

N.A. not applicable

Table S2. FTIR bands characterizing different oxidation states of the H-cluster from various [FeFe] hydrogenases.

State	Name	CN	t-CO	μ-CO	Ref.
H_{ox}	*Tm*HydABC	2090, 2076	1964, 1939	1802	This work
	*Tm*HydA	2090, 2076	1964, 1939	1802	This work
	*Tm*HydABC	2080, 2075	1965, 1939	1800	[14]
	*Tm*HydS	2088, 2079	1971, 1947	1806	[4]
	*Cr*HydA1	2088, 2072	1964, 1940	1800	[15]
	*Dd*HydAB	2093, 2079	1965, 1940	1802	[16]
	*Cp*I	2086,2072	1971, 1940	1802	[17]
H_{red}	*Cr*HydA1	2083, 2070	1962, 1932	1791	[15, 18]
	*Dd*HydAB	2088, 2072	1964, 1934	1789	[19]
$H_{red}H^{+}$	*Tm*HydABC	2075, 2037	1956, 1919, 1887	-	This work
	*Tm*HydA	2075, 2037	1956, 1919, 1887	-	This work
	*Cr*HydA1	2071, 2032	1968, 1914, 1891	-	[18]
	*Dd*HydAB	2079, 2041	1965, 1915, 1894	-	[16, 19]
	*Cp*I	2071, 2053	1962, 1915, 1899	-	[20]
	*Ca*I	2055,2040	1921,1899	1801	[21]
H_{ox}-CO	*Tm*HydABC	2092, 2085	2008, 1970, 1961	1807	This work
	*Tm*HydA	2092, 2085	2008, 1970, 1961	1807	This work
	*Tm*HydABC	-	2009, 1971, 1964	1808	[14]
	*Tm*HydS	2090	2016, 1973, 1964	1805	[4]
	*Cr*HydA1	2092, 2084	2013, 1970, 1964	1810	[15]
	*Dd*HydAB	2096, 2088	2016, 1971, 1963	1810	[16]
	*Cp*I	2095, 2077	2017, 1974, 1971	1810	[17]

Table S3. Collection of g-values of different oxidation states of the H-cluster from various [FeFe] hydrogenases.

State	Name	g1	g2	g3	Ref.
H_{ox}	*Cr*HydA1	2.104	2.042	1.998	[22]
	*Dd*HydAB	2.100	2.040	1.998	[3, 23]
	*Cp*HydA1	2.097	2.039	1.999	[24]
	*Tm*HydS	2.113	2.045	2.001	[4]
	*Tm*HydABC	2.102	2.044	1.998	This work
	*Tm*HydA	2.103	2.045	1.999	This work
H_{ox}-CO	*Cr*HydA1	2.054	2.009	2.009	[22]
	*Dd*HydAB	2.065	2.007	2.001	[23]
	*Cp*HydA1	2.072	2.006	2.006	[24]
	*Tm*HydS	2.047	2.018	2.007	[4]
	*Tm*HydABC	2.064	2.008	2.005	This work
	*Tm*HydA	2.062	2.011	2.006	This work

Table S4. The g-values (g1, g2, g3), and the g-strain parameters (gS1, gS2, gS3) used to simulate the EPR spectra in Fgure 5B and 6B including assigments of the components to specific species

Simulation parameters of *Tm*HydABC:

Treatment	Component	g1	g2	g3	gS1	gS2	gS3	weight
**NAD^+**	1 (H_{ox})	2.102	2.044	1.998	0.006	0.005	0.006	1.37
	2 (H_{ox}-CO)	2.064	2.008	2.005	0.017	0.009	0.008	0.50
	3 [2Fe-2S]	2.013	1.950	1.917	0.007	0.018	0.019	2.18
**CO-purging**	1 (H_{ox}-CO)	2.064	2.008	2.005	0.014	0.006	0.008	3.01
	2 [2Fe-2S]	2.014	1.950	1.916	0.007	0.020	0.016	3.60

Simulation parameters of *Tm*HydA:

Treatment	Component	g1	g2	g3	gS1	gS2	gS3	weight
**Thionine**	1 (H_{ox})	2.103	2.045	1.999	0.006	0.006	0.006	2.05
	2 (H_{ox}-CO)	2.062	2.011	2.006	0.015	0.008	0.008	0.70
	3 [2Fe-2S]	2.005	1.949	1.918	0.013	0.017	0.014	5.48
**CO-purging**	1 (H_{ox}-CO)	2.062	2.011	2.006	0.016	0.006	0.005	1.64
	2 [2Fe-2S]	2.018	1.947	1.917	0.014	0.019	0.016	6.17

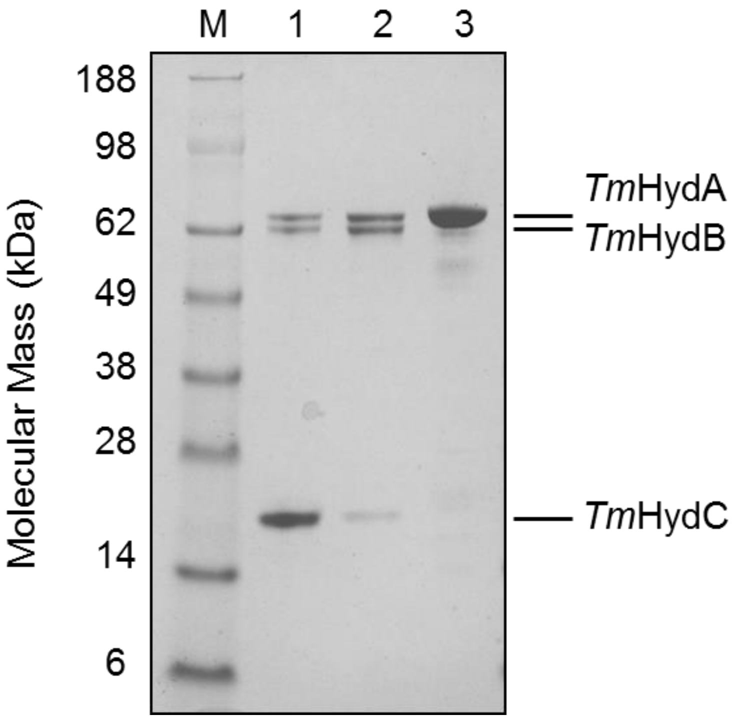

Fig. S1. SDS PAGE analysis of recombinantly overexpressed and purified apo-*Tm*HydABC and apo-*Tm*HydA. Samples of the protein molecular weight markers (lane M, Seeblue Plus 2 prestained protein standard, Thermo Fisher), apo-*Tm*HydABC after affinity chromatography (lane 1) and gel filtration chromatography (lane 2), and apo-*Tm*HydA after affinity chromatography (lane 3) were loaded onto a 4-12% Bis-Tris precast gel and electrophoresis was performed at 200 V, 60 mA for 1.0 h at room temperature.

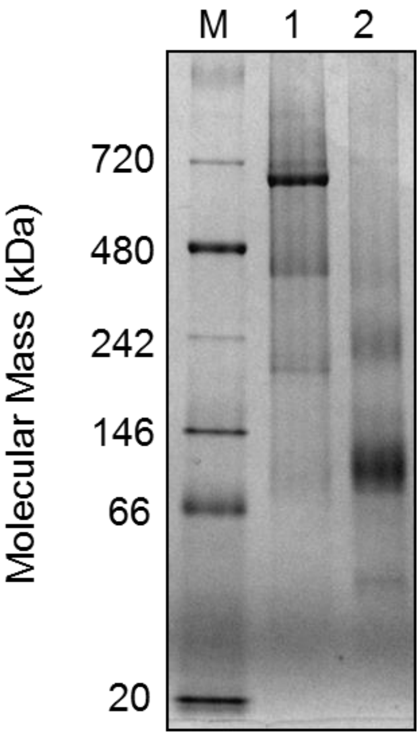

Fig. S2. Native PAGE analysis of purified apo-*Tm*HydABC and apo-*Tm*HydA. Samples of the protein molecular weight markers (lane M, NativeMark™ unstained protein standards, Thermo Fisher), apo-*Tm*HydABC after gel filtration chromatography (lane 1) and apo-*Tm*HydA (lane 2) after affinity chromatography were loaded onto a 4-16% NativePAGE™ Bis-Tris precast gel and electrophoresis was performed at constant voltage (150 V) for 1.5 h at room temperature.

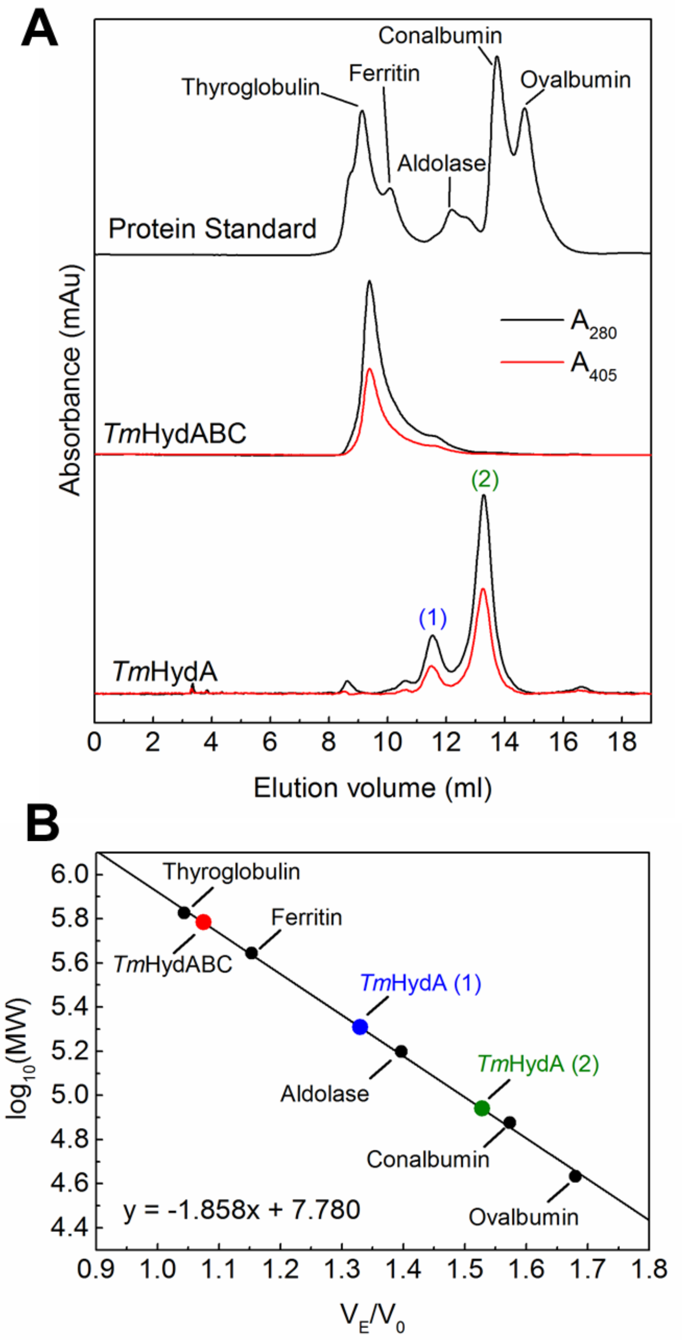

Fig. S3. Analytical size exclusion chromatography of apo-*Tm*HydABC and apo-*Tm*HydA. (A) Chromatographic traces for a mixture of protein standards (Thyroglobulin 669 kDa, Ferritin 440 kDa, Aldolase 158 kDa, Conalbumin 75 kDa, and Ovalbmin 43 kDa), *Tm*HydABC, and *Tm*HydA are shown. These traces were recorded at room temperature, by injecting 100 µl of each sample onto a 24 ml Superdex 200 increase 10/300 GL column (GE Heathcare), pre-equilibrated in 100 mM Tris-HCl, 150 mM NaCl, pH 8 buffer. The samples were eluted at 0.5 ml/min flowrate. The elution profile at pH 8 shows a single elution peak for *Tm*HydABC indicating that it forms a single oligomeric form at this pH. In contrast, the elution profile of *Tm*HydA shows two peaks, indicating that the protein exists in two oligomeric states. (B) The calibration curve of the protein standards is shown. V_E and V_0 represents the elution volume and void volume respectively. The red circular marker indicates V_E of *Tm*HydABC, while blue and green markers indicate V_E of *Tm*HydA.

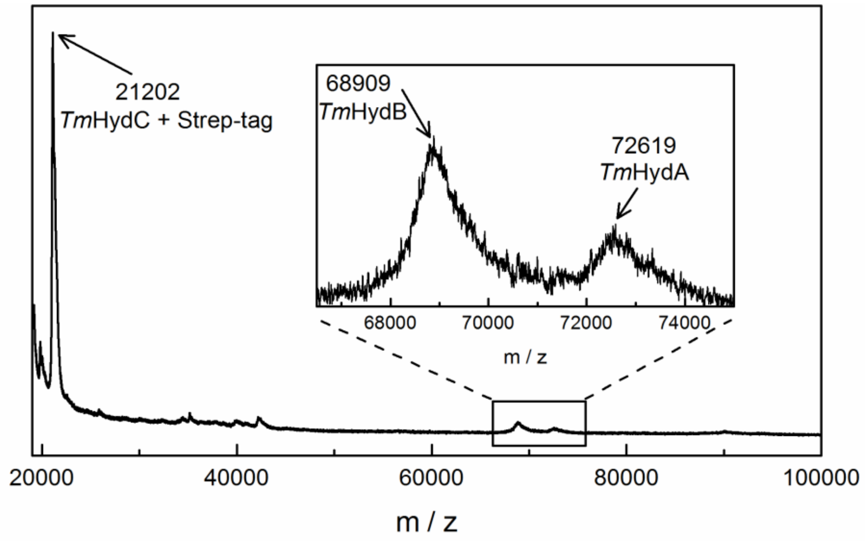

Figure S4. MALDI TOF-MS spectrum of apo-*Tm*HydABC. The purified apo-*Tm*HydABC was mixed with sinapic acid matrix and the mixture was air-dried before measuring the spectrum with a Voyager-DE PRO MALDI-TOF-System (Applied Biosystems).

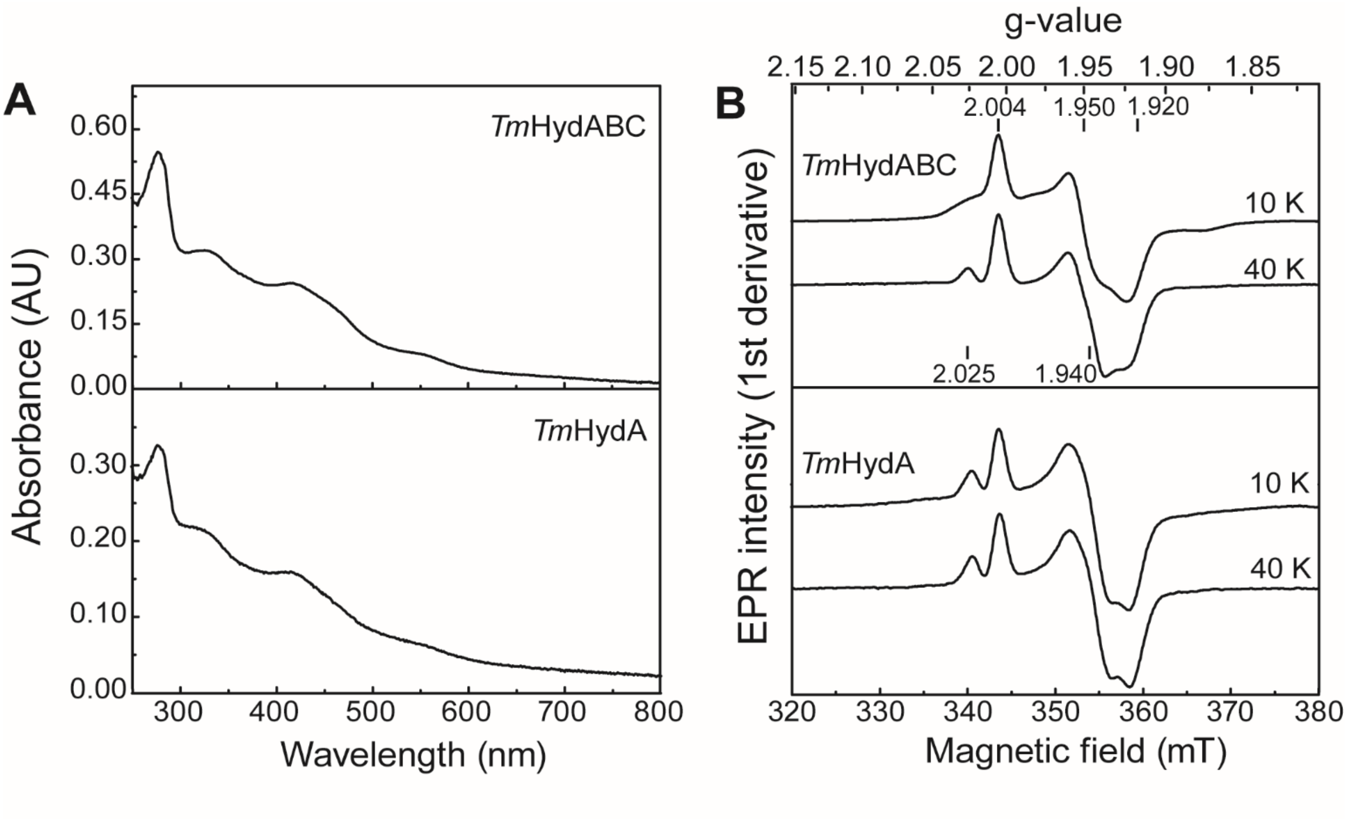

Figure S5. Spectroscopic characterization of apo-*Tm*HydABC and apo-*Tm*HydA. (A) UV-visible spectra of the proteins were measured in 0.1 M Tris-HCl pH 8, 0.15 M NaCl buffer at room temperature. (B) CW X-band EPR spectra of the apo-proteins (reduced with sodium dithionite) were measured in the same buffer at 10 K (0.01 mW microwave power) and 40 K (1 mW microwave power), respectively.

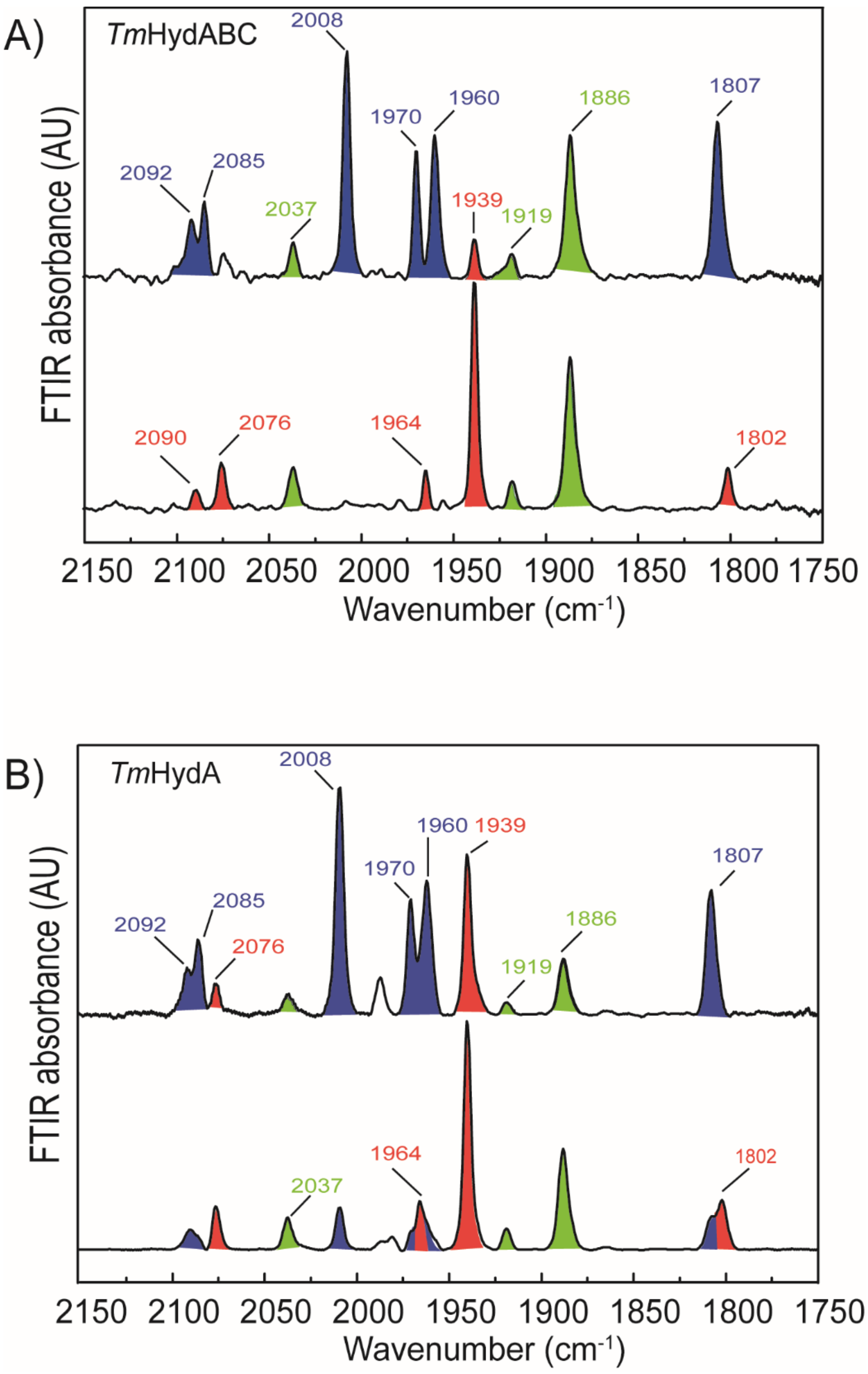

Figure S6. FTIR spectra of (A) *Tm*HydABC and (B) *Tm*HydA after artificial maturation. In both (A) and (B), the top panels show the FTIR spectra of the proteins just after removal of the excess $[2Fe]^{adt}$ complex and the bottom panels show FTIR of the same protein samples after overnight shaking inside a glove box (2% H_2, 98% N_2). Peaks colored red are assigned to the H_{ox} state, peaks colored green are assigned to the $H_{red}H^+$ state and peaks colored blue are assigned to the H_{ox}-CO state.

```
Tmari0929   ------------------------------------M---KVRVDADACIG-CG--VCENLCP 21
Tmari1182   --------------------------------MAKNW---YPVIDYGKCTG-CL--TCVNFCP 25
Tmari1297   ------------------------MPW-----------------VNSKCVG-CG--NCVKVCP 19
Tmari1541   --------------------MRIEDKLYLNRYRTDEENPHLKIKDESICAEKCSDRPCVSCCP 43
Tmari1825   MAEAKNAPLIGKDALGREVRDLSKVPWWGVDRKEIEW---YPTIDYDKCVT-CG--ICFVTCG 57


Tmari0929   DVFQLGDDGKAKVLQPETDLPCAKDA--ADSCPTGAISVEE-------------------   60
Tmari1182   HGVYTA--ENGKPKVSNPDACVEFCKGCQKICPAGAINYSAEVSADG-------------   70
Tmari1297   VEGAIR-IENGKA-VIDNYKCIR-CGKCFDACPVGAIRPNYENPALRGMGRGFGRGLGRG   76
Tmari1541   ADVYEWT-ESGM--EVKFEGCLE-CGTCRIVCPFGNIEWNYPRGNYGVL-YKFG------   92
Tmari1825   RRVFDFDKKEGKVIVARPYNCMVACQTCMNLCPTGAISFPDASYIKKLVAQN--KIVKKA  115


Tmari0929   --------------------------------   60
Tmari1182   --------------------------------   70
Tmari1297   FGRGMGRGYGRGRGRW--DEF-----------   95
Tmari1541   --------------------------------   92
Tmari1825   FE--------IIKPLLAEDHLSPKETETKPEP  139
```

Figure. S7. Sequence alignment of all five ferredoxins found in the genome of *T. maritima*. Cysteine residues are highlighted in yellow. Except *Tm*Fd1 (Tmari0929), the other four ferredoxins contain 2x [4Fe-4S] clusters. Alignments were created in Clustal Omega with sequences obtained from the Uniprot website (accession codes: Tmari0929: G4FF63, Tmari1182: R4NS93, Tmari1297: R4NSM3, Tmari1541: R4NRM2, and Tmari1825: R4P208).

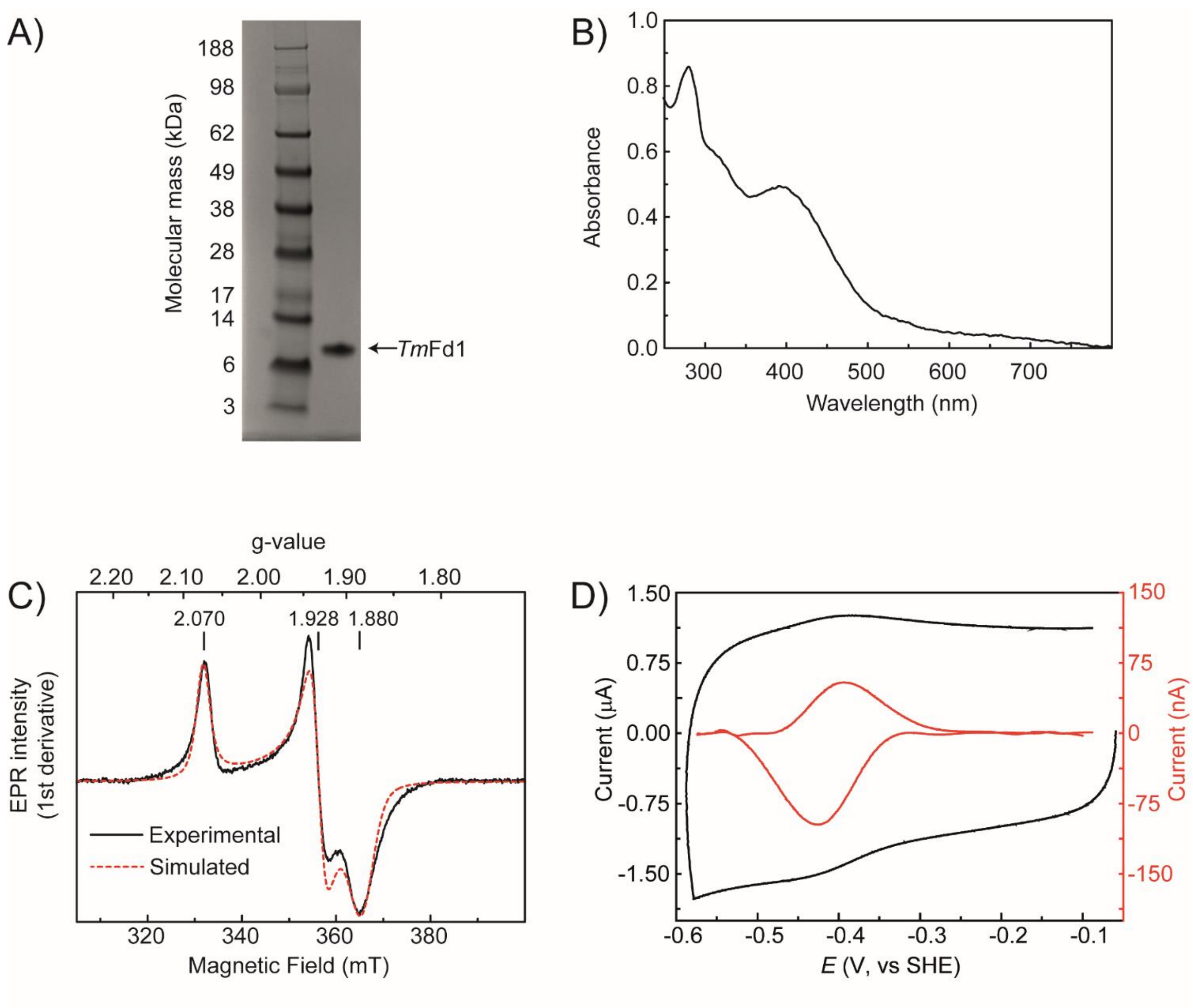

Figure S8. Characterization of recombinant *Tm*Fd1. (A) SDS-PAGE analysis of purified *Tm*Fd1. The purified protein and the molecular weight marker (Seeblue plus 2 prestained protein standard, Thermofisher) were loaded onto a 4-12% Bis-Tris precast gel and electrophoresis was performed at -200 V, 60 mA for 1 h 20 min at room temperature. (B) UV-visible spectrum of purified *Tm*Fd1 in 0.1 M Tris-HCl pH 8, 0.15 M NaCl buffer at room temperature. (C) CW-X-band EPR spectrum of sodium dithionite reduced *Tm*Fd1 measured in the same buffer at 10 K and 0.1 mW microwave power (shown in solid black line). The spectrum was simulated using EasySpin (trace shown in dotted red line) to extract the g-values of the [4Fe-4S] cluster of *Tm*Fd1. (D) Cyclic-voltammetry of the *Tm*Fd1 non-turnover signals measured on a PGE electrode (2 mm diameter) measured at 25 °C, under N_2 atmosphere, and in pH 8 buffer mix. The midpoint potential of *Tm*Fd1 extracted from the above measurement is -410 mV, vs SHE.

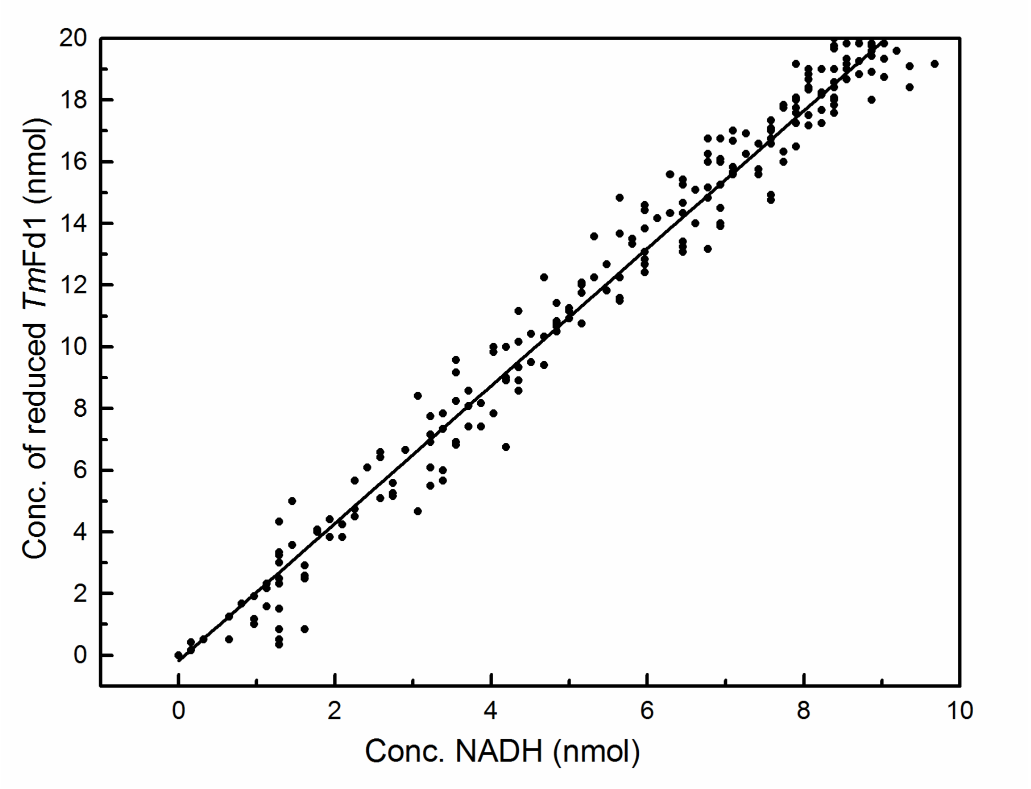

Figure S9. Estimation of the stoichiometry of NAD^+ and *Tm*Fd1 reduction during the electron bifurcation reaction with *Tm*HydABC. The concentration of reduced *Tm*Fd1 and NADH were calculated using the absorbance at 430 and 340 nm, respectively during *Tm*HydABC catalyzed electron bifurcation. The reaction was started by adding 0.5 mM NAD^+ to a mixture of ≈700 ng *Tm*HydABC, 50 µM FMN and 35 µM *Tm*Fd1.

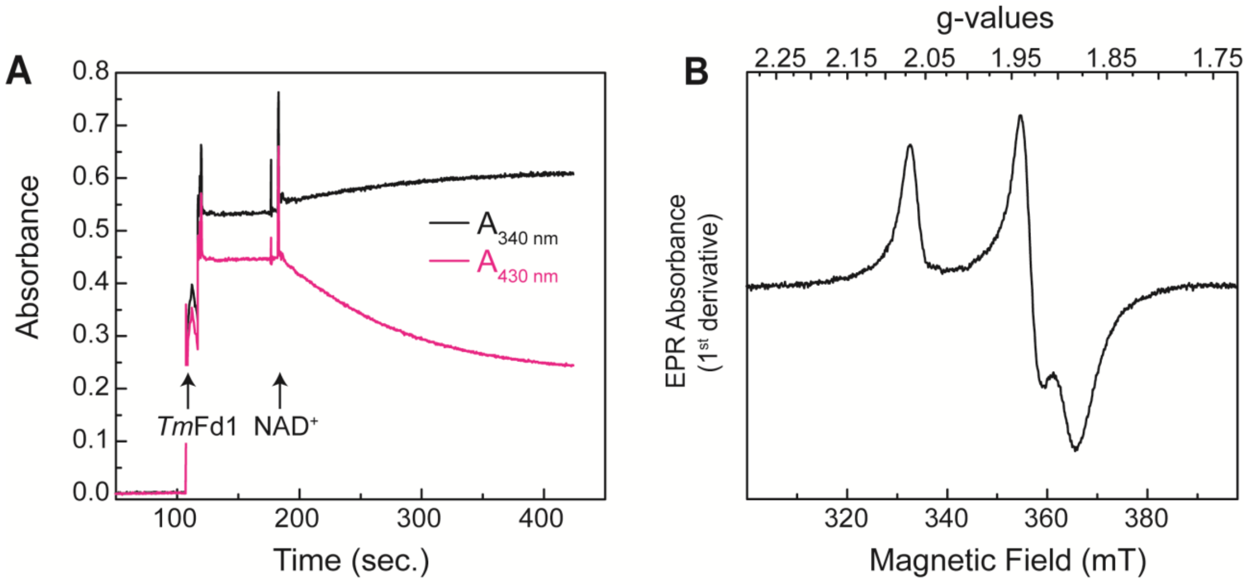

Figure S10. Reduction of NAD^+ and *Tm*Fd1 by *Tm*HydABC in the absence of added FMN. (A) Reduction of *Tm*Fd1 and NAD^+ were monitored at 70 °C under an 100% H_2 atmosphere. To a 1 ml reaction mixture (200 mM potassium phosphate buffer, pH 8) containing ≈680 ng *Tm*HydABC, 35 µM *Tm*Fd1 was added (indicated by the first arrow). To the same reaction mix, 0.5 mM NAD^+ was added (indicated by the second arrow). (B) The reduction of *Tm*Fd1 during the reaction was also confirmed by EPR spectroscopy. 200 µl of the assay mix was transferred to an X-band EPR tube and frozen to measure the EPR spectrum at 10 K and 0.2 mW power.

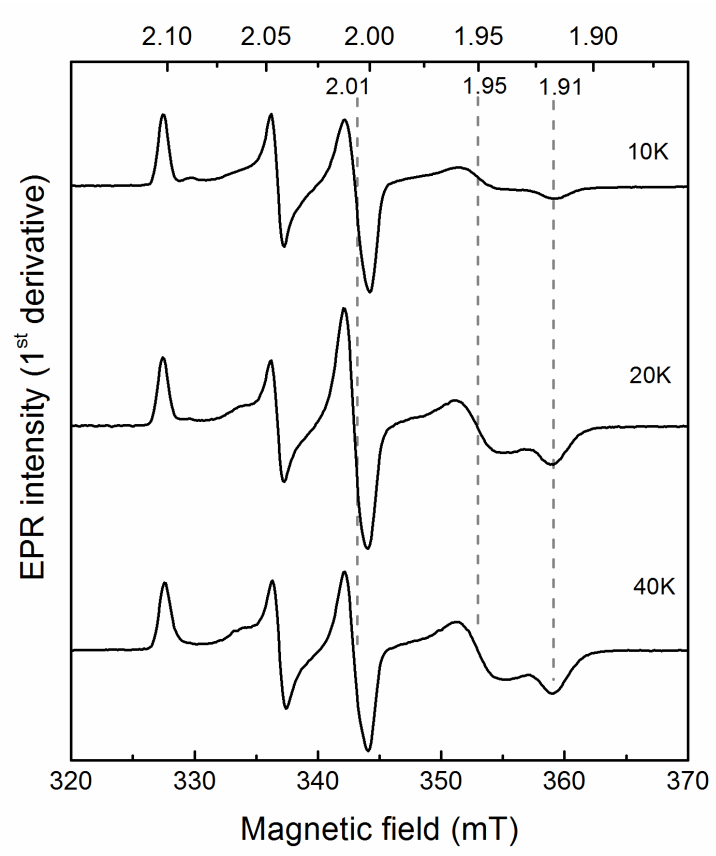

Figure S11. CW X-band EPR spectra of an NAD^+ oxidized *Tm*HydABC sample at T = 10 K (0.1 mW microwave power), 20 K (0.1 mW microwave power) and 40 K (1 mW microwave power).

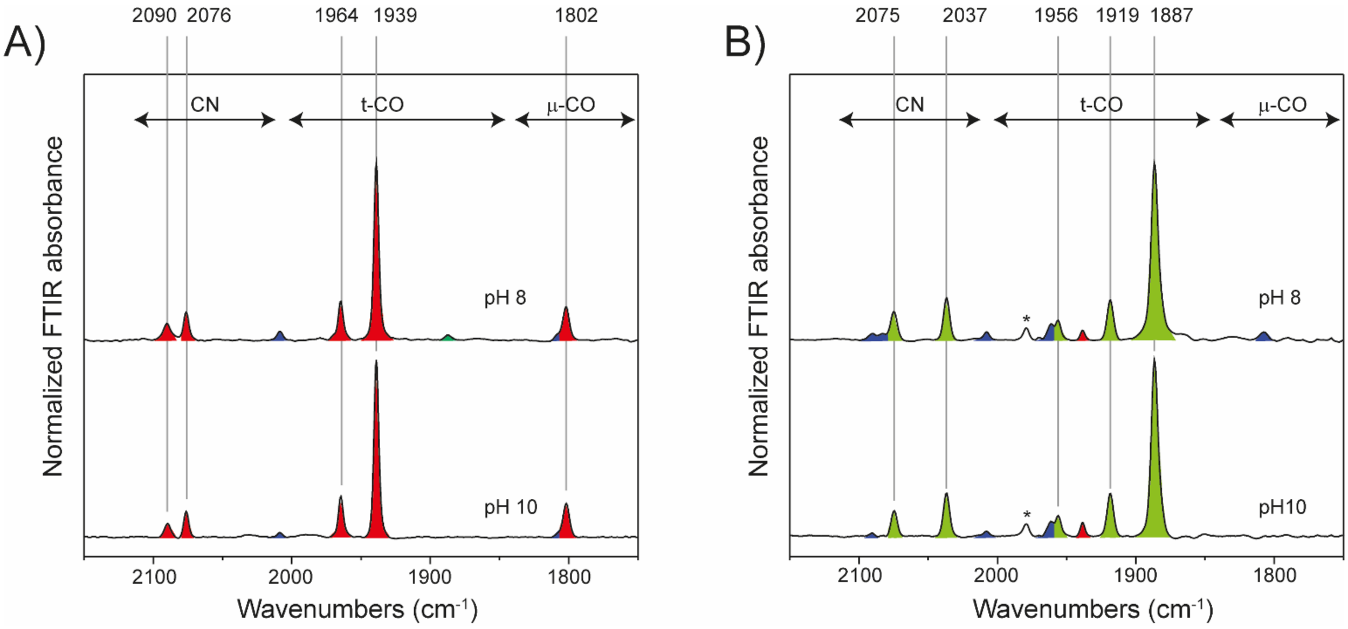

Figure S12. FTIR spectra of *Tm*HydABC at pH 8 and 10. (A) FTIR spectra of 400 μM *Tm*HydABC oxidized with 20 mM NAD^+ in 100 mM Tris-HCl buffer, 100 mM KCl, pH 8 and 50 mM Glycine/MES/HEPES, 100 mM KCl buffer pH 10. (B) FTIR spectra of the same concentration of *Tm*HydABC reduced with 20 mM sodium dithionite in 100 mM Tris-HCl buffer, 100 mM KCl, pH 8 and 50 mM Glycine/MES/HEPES, 100 mM KCl buffer pH 10.

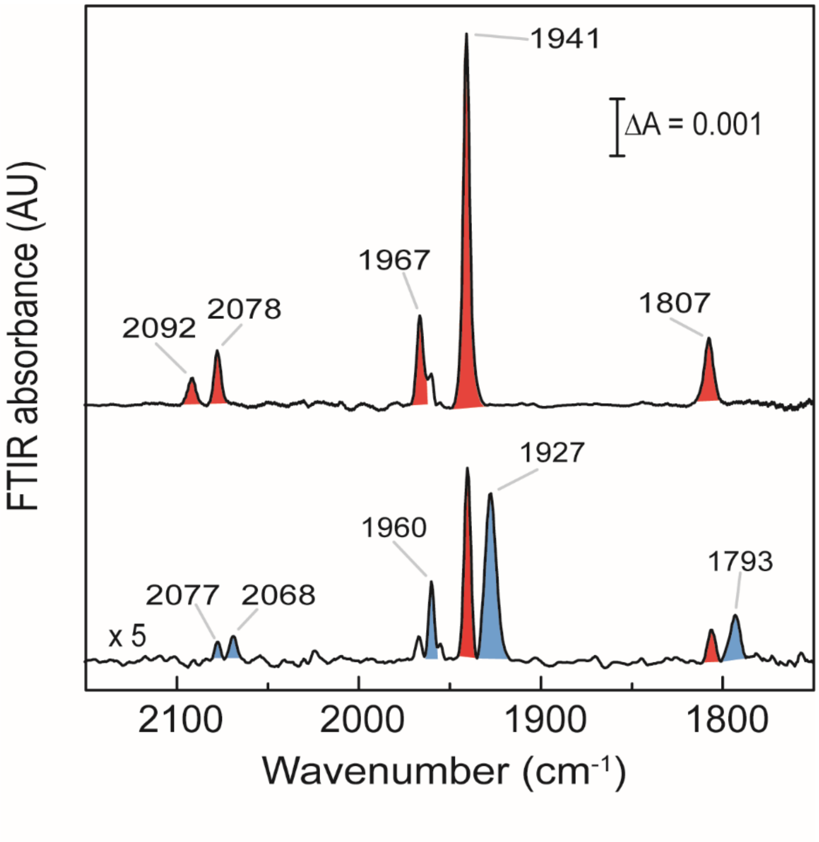

Figure. S13. FTIR spectra of *Tm*HydABC maturated with $[2Fe]^{PDT}$. The protein was incubated with the synthetic complex (1:3) in 0.1 M Tris-HCl, 0.15 M NaCl, pH 8 for 1 h and then the excess complex was removed by desalting through the PD10 column. The top panel represents FTIR spectra of as isolated *Tm*HydABC^{PDT} sample and the lower panel shows the FTIR spectra of NaDT reduced (1:10) *Tm*HydABC^{PDT}. Both the spectra were recorded at room temperature.

Reference

1. Kuchenreuther JM, Grady-Smith CS, Bingham AS, George SJ, Cramer SP, Swartz JR (2010) PLoS One 5:e15491
2. Tartoff K, Hobbs C (1987) Bethesda Res. Lab. Focus 9 12
3. Birrell JA, Wrede K, Pawlak K, Rodríguez-Maciá P, Rüdiger O, Reijerse EJ, Lubitz W (2016) Isr. J. Chem. 56:852-863
4. Chongdar N, Birrell JA, Pawlak K, Sommer C, Reijerse EJ, Rudiger O, Lubitz W, Ogata H (2018) J. Am. Chem. Soc. 140:1057-1068
5. Verhagen MF, O'Rourke T, Adams MW (1999) Biochim. Biophys. Acta 1412:212-229
6. Huberman A, Perez C (2002) Anal. Biochem. 307:375-378
7. Aliverti A, Curti B, Vanoni MA (1999) Methods Mol. Biol. 131:9-23
8. Esselborn J, Lambertz C, Adamska-Venkatesh A, Simmons T, Berggren G, Noth J, Siebel J, Hemschemeier A, Artero V, Reijerse E, Fontecave M, Lubitz W, Happe T (2013) Nat. Chem. Biol. 9:607-609
9. Li HX, Rauchfuss TB (2002) J. Am. Chem. Soc. 124:726-727
10. Greene BL, Schut GJ, Adams MWW, Dyer RB (2017) ACS Catal. 7:2145−2150
11. Schut GJ, Adams MW (2009) J. Bacteriol. 191:4451-4457
12. Blamey JM, Mukund S, Adams MW (1994) FEMS Microbiol. Lett. 121:165-169
13. Maiocco SJ, Arcinas AJ, Booker SJ, Elliott SJ (2019) Protein Sci. 28:257-266
14. Greene BL, Schut GJ, Adams MWW, Dyer RB (2017) ACS Catal. 7:2145-2150
15. Silakov A, Kamp C, Reijerse E, Happe T, Lubitz W (2009) Biochemistry 48:7780-7786
16. Roseboom W, De Lacey AL, Fernandez VM, Hatchikian EC, Albracht SP (2006) J. Biol. Inorg. Chem. 1:102-118
17. Chen Z, Lemon BJ, Huang S, Swartz DJ, Peters JW, Bagley KA (2002) Biochemistry 41:2036-2043
18. Sommer C, Adamska-Venkatesh A, Pawlak K, Birrell JA, Rüdiger O, Reijerse EJ, Lubitz W (2017) J. Am. Chem. Soc. 139:1440-1443
19. Rodríguez-Maciá P, Pawlak K, Rüdiger O, Reijerse EJ, Lubitz W, Birrell JA (2017) J. Am. Chem. Soc. 139:15122-15134
20. Winkler M, Senger M, Duan J, Esselborn J, Wittkamp F, Hofmann E, Apfel UP, Stripp ST, Happe T (2017) Nat. Commun. 8:16115
21. Ratzloff MW, Artz JH, Mulder DW, Collins RT, Furtak TE, King PW (2018) J Am Chem Soc 140:7623-7628
22. Esselborn J, Lambertz C, Adamska-Venkates A, Simmons T, Berggren G, Noth J, Siebel J, Hemschemeier A, Artero V, Reijerse E, Fontecave M, Lubitz W, Happe T (2013) Nat. Chem. Biol. 9:607-609
23. Albracht SP, Roseboom W, Hatchikian EC (2006) J. Biol. Inorg. Chem. 11:88-101
24. Bennett B, Lemon BJ, Peters JW (2000) Biochemistry 39:7455-7460
